# The IFN-γ response cycle: decoupling tumor killing from evolutionary selection in cancer immunotherapy

**DOI:** 10.3389/fimmu.2026.1954319

**Published:** 2026-10-01

**Authors:** Baiyu Wang, Delong Zhao, Yuehan Ma, Chengyu Sui

**Affiliations:** 1 Department of Anesthesiology, Jilin Cancer Hospital, Changchun, China; 2 Department of Ultrasound, Jilin Cancer Hospital, Changchun, China; 3 First Surgery Department of Colorectal, Gastric, and Abdominal Tumors, Jilin Cancer Hospital, Changchun, China; 4 Department of Anesthesiology, Women’s Hospital, School of Medicine, Zhejiang University, Jilin Hospital, Changchun, China

**Keywords:** adaptive resistance, cancer immunotherapy, immune checkpoint blockade, interferon-γ, tumor immunoediting

## Abstract

Interferon-γ (IFN-γ) confronts the field with an apparent contradiction that is better understood as a causal consequence: the cytokine most central to antitumor immunity is also a principal driver of immune escape, and recombinant IFN-γ has both helped and harmed patients. We argue that this duality is not paradoxical but expected, escape is, in large part, the predictable evolutionary outcome of effective IFN-γ–mediated pressure. The prevailing “two faces” framing, which separates antitumor and protumor effects by dose and duration, names this discrepancy without explaining what couples its halves. Here, we reframe IFN-γ as a time-ordered IFN-γ Response Cycle: a recurring causal motif, productive execution, a self-limiting turn, and selectable resistance, running around one tunable IFNGR–JAK–STAT1–IRF1 backbone. We use “cycle” deliberately and in a restricted sense: not to imply that an individual tumor clone oscillates between sensitive and resistant states, but that this execute–feedback–select motif recurs across a tumor’s heterogeneous clonal population and across successive rounds of immune pressure and therapy. Its reversible arc, adaptive feedback and non-genetic gating, can be therapeutically reopened, whereas genetic loss of the pathway constitutes a one-way exit from the cycle rather than a return within it. Its behavior is set less by signal magnitude than by decoding, which cells read the cytokine, at what intensity, in what spatial arrangement, and over what kinetics. Synthesizing evidence across tumor killing, antigen presentation, the cellular decoding network, adaptive and chronic feedback, genetic escape and induced phenotypic adaptation, therapeutic re-engineering, and biomarkers, we argue that resistance is largely manufactured by the very signaling that mediates control: by eliminating sensitive clones, IFN-γ selects for resistant clones. This recasts IFN-γ as a self-defeating selective force and reframes the therapeutic goal as decoupling killing from selection, preserving execution while severing the feedback and selection arc. IFN-γ biomarkers currently report the presence and dominance of an inflammatory, IFN-γ-driven state rather than a defined position within the cycle; longitudinal, spatial, and compartment-resolved measurements may eventually permit a finer classification.

## 1 Introduction

Interferon-γ (IFN-γ) has long been recognized as having context-dependent and apparently opposing roles in cancer (1–3). By promoting type 1 immune responses, cytotoxic effector functions, and antigen presentation, IFN-γ contributes substantially to antitumor immunity; however, it can also induce mechanisms of immune escape, and clinical studies of recombinant IFN-γ have reported both beneficial and adverse outcomes (1–3). This review argues that these opposing effects need not be viewed as an inexplicable paradox. Sustained IFN-γ exposure can itself induce immunoregulatory programs that favor tumor immune evasion (4), while effective antitumor immune pressure can also favor the emergence or outgrowth of tumor-cell populations with impaired IFN-γ signaling (5, 6). Consistent with the importance of this pathway, defects in tumor-cell IFN-γ signaling have been associated with resistance to both PD-1 and CTLA-4 checkpoint blockade (6, 7). Reconciling these observations as two dose- and duration-separated “faces” of one molecule describes the discrepancy but leaves its mechanistic basis unresolved (Section 2).

An alternative reading is temporal: not independent properties but differentially weighted outputs, with their balance set by the duration and magnitude of exposure (4). Chronic exposure similarly imposes lineage-dependent, incompletely reversible adaptive states that favor tumor persistence (8). The intensity and timing of exposure dictate whether myeloid precursors acquire competent or defective antigen-presenting function (9). The output also depends on the cellular compartment that decodes the signal, with tumor-intrinsic and immune-cell-intrinsic sensing generating opposing programs (10).

The pathway runs on a canonical backbone, IFNGR1/2, JAK1/JAK2, and STAT1 homodimers acting at gamma-activated sequence (GAS) elements to induce IRF1, with STAT2- and STAT3-dependent branches operating as context-dependent modifiers rather than core components, with STAT3 frequently antagonizing STAT1 output (11). Its bifurcation sits at IRF1, a shared upstream node acting on the antigen-presentation arm through the IRF-E of the bidirectional TAP1/PSMB9 promoter (12), with STAT1 at CIITA promoter IV (13), and on IFN-γ–induced PD-L1 in melanoma cells (11); therefore, a single node feeds both effector and inhibitory outputs. Consistent with, though not directly demonstrated by, this architecture, pretreatment nuclear IRF1 expression was more strongly associated with objective response and progression-free survival than PD-L1 expression in a retrospective cohort of patients with metastatic melanoma receiving anti-PD-1-based therapy (14). More broadly, IFN-γ pathway output varies according to the tumor and cellular context: pan-cancer TCGA analysis identified opposite prognostic associations across individual tumor types, whereas single-cell RNA sequencing revealed heterogeneous pathway distribution and regulation among stromal, immune, and malignant cells (15).

This context-dependent architecture creates selective asymmetry: IFN-γ signaling that supports tumor control can, under sustained immune pressure, favor the expansion of tumor cells that no longer respond to it (5). Acquired resistance to anti-PD-1 therapy was associated with loss-of-function JAK1 or JAK2 mutations and, in another patient, a truncating B2M mutation (6). Although these mutations were not detected in the pretreatment samples, rare pre-existing subclones cannot be excluded (6). In heterogeneous tumors, CD8^+^ T-cell-derived IFN-γ selectively expands IFNGR2-mutant cells, whereas PD-L1 supplied by neighboring wild-type cells protects them from immune elimination (5). IFN-γ also induces BCAT1-dependent conversion of non-stem cancer cells into stem-like states, providing a non-genetic mechanism of adaptation (16). In a phase 1 trial, nivolumab plus IFN-γ was tolerable and increased PD-L1 expression on circulating monocytes, particularly at higher doses; however, its relationship with intratumoral PD-L1 expression or clinical response remained unclear (17).

Here, we integrate evidence on tumor killing, antigen presentation, signaling feedback, genetic escape, induced phenotypic adaptation, therapeutic intervention, and biomarkers into a single IFN-γ Response Cycle. Clinical resistance evidence derives mainly from melanoma cohorts, whereas mechanistic context dependence is supported largely by pan-cancer analyses and preclinical models (5–7, 15, 18). Three gaps remain: clonal selection has not been prospectively tracked in patients in these studies (5–7); validated thresholds linking exposure and cellular context to outcome are undefined (5, 15, 18); and IFN-γ signatures remain correlational predictors whose treatment-related decline may have context-dependent interpretations (19, 20). Parts I–VI trace this cycle from tumor control mechanisms through adaptation, therapeutic intervention points, and biomarkers. The central question is whether therapy can preserve IFN-γ-mediated tumor control without enabling adaptive selection.

## 2 Part I. foundations: the dual-role problem and the signal

### 2.1 The dual-role problem: from “two faces” to one cycle

The “two faces” framing separates IFN-γ’s antitumor and protumor effects chiefly by dose and duration, treating them as coexisting properties of a single molecule. Its limitation is that it supplies no coupling mechanism; it does not specify why one signal should yield one output and then the other, nor what governs the transition between them. A complementary reading is that the two halves are not independent properties but differentially weighted outputs of a single signal, whose relative contribution changes with the duration and magnitude of the exposure. In a model permitting sustained exposure, transient IFN-γ was inert, sustained low-level signaling promoted hepatoma, mammary carcinoma, and melanoma, and sustained high-level signaling remained antitumor; the protumor state was accompanied by induction of PD-L1, PD-L2, CTLA-4, and Foxp3 and abrogated by checkpoint-ligand blockade (4). Such reversibility is consistent with suppression arising as a feedback consequence of the signaling that initially mediates control, rather than an independent property, a logic anticipated by the proposal that tumor cells sensing CTL-derived IFN-γ preemptively raise PD-L1 (2).

Checkpoint blockade renders this coupling explicit in a compartment-specific way. Tumor-intrinsic IFN-γ signaling establishes inhibitory programs involving PD-L1-associated T cell exhaustion and impaired TRAIL-dependent innate cytotoxicity. In contrast, IFN-γ activity within immune cells sustains adaptive tumor rejection in antigenic tumors or promotes innate effector maturation when neoantigen expression or MHC-I is lost (10). Thus, productive and self-limiting programs do not appear as alternatives but as concurrent outputs of one signal, the balance of which shifts over time. These observations are more coherently read not as two static faces but as successive phases of one causal sequence: productive activation, feedback regulation, and adaptive resistance follow in order rather than coexisting as fixed properties. We formalize this sequence as the IFN-γ Response Cycle. Two clarifications define what this term does and does not claim. First, the recurring element is the causal motif, productive killing elicits counter-regulatory feedback, which selects for reduced responsiveness, not the state of any single clone; individual clones do not oscillate. The motif “cycles” because a genetically and phenotypically heterogeneous tumor continually presents the immune system with still-responsive cells, and because checkpoint blockade and re-sensitizing agents can relieve feedback and reopen the reversible portion of the arc (Section 12). Second, the loop is bounded: reversible adaptation (metabolic and epigenetic gating, phenotypic plasticity; Sections 10, 11.3) can return a cell toward responsiveness, but genetic loss of the IFNGR–JAK–STAT or antigen-presentation machinery (Section 11.1) is effectively irreversible and marks a one-way exit from the cycle into fixed resistance and progression. The cycle therefore describes tumor–immune coevolution at the level of the population over time, not a reversible oscillation of one lesion (**Figure 1**).

**Figure 1:**
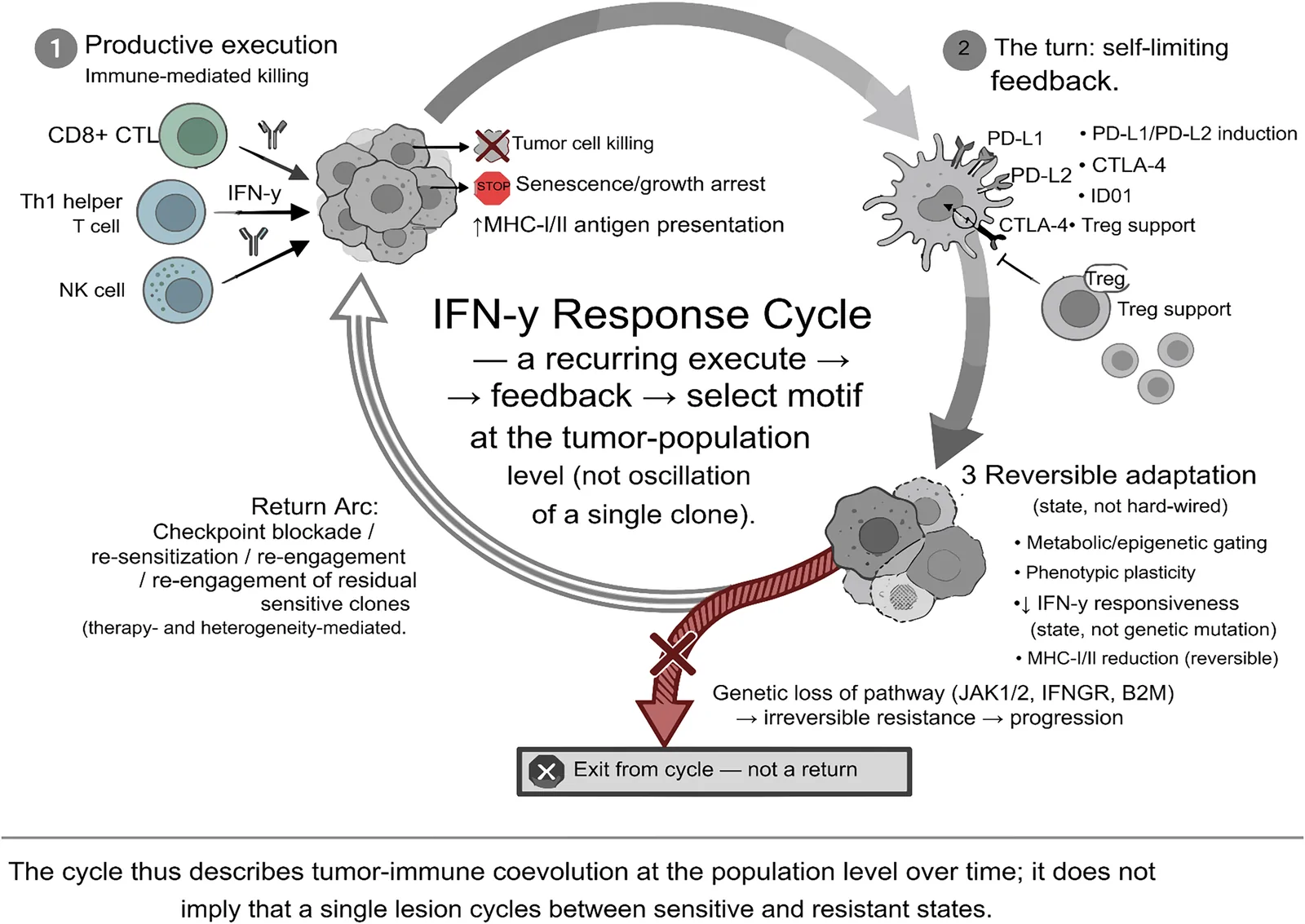
The IFN-γ response cycle: a recurrent, bounded causal motif, not an oscillation of individual clones. Productive IFN-γ signaling on a sensitive tumor population (Node 1: killing, senescence, antigen presentation) elicits self-limiting counter-regulation (Node 2: PD-L1/PD-L2, CTLA-4, IDO1, Treg support), which selects for reduced responsiveness (Node 3: reversible metabolic/epigenetic gating and phenotypic plasticity). The loop is closed not by spontaneous re-sensitization of a resistant clone but by therapy and tumor heterogeneity, checkpoint blockade and re-sensitizing agents relieve feedback, and residual or newly arising sensitive clones re-enter productive signaling (return arc). Critically, genetic loss of the IFNGR–JAK–STAT or antigen-presentation machinery is drawn as a one-way exit from the cycle (JAK1/2, IFNGR, B2M loss → irreversible resistance → progression), consistent with the observation that such lesions do not revert. The cycle thus describes tumor–immune coevolution at the population level over time; it does not imply that a single lesion cycles between sensitive and resistant states.

This framing differs from the conventional account of adaptive and acquired resistance in one specific respect. Linear models treat adaptive resistance (e.g., feedback PD-L1 induction) and acquired resistance (e.g., JAK1/2 or B2M loss) as separate stages arranged end-to-end, each explaining a different clinical failure (4). The cycle makes a stronger causal claim: that the same productive signaling responsible for tumor control is also the proximate cause of the feedback and the selective pressure that generate resistance, so efficacy and escape are not sequential accidents but two outputs of one engagement (5, 7). The practical corollary is testable and distinguishes the two views: if resistance were merely a later, independent stage, maximizing IFN-γ should uniformly improve outcomes; if resistance is manufactured by the signaling itself, then, beyond a threshold—additional undifferentiated IFN-γ should accelerate selection, and benefit should instead come from uncoupling execution from feedback (Section 12) (4). Existing data are consistent with, though do not yet prove, the latter: in a phase 1 trial, recombinant IFN-γ added to nivolumab raised PD-L1 on circulating monocytes with increasing dose and duration, without evidence of improved response (17).

Two readings of this ordering are possible, and the distinction is consequential. Under a strictly sequential reading, productive activation, feedback regulation, and adaptive resistance would be discrete states that a tumor occupies one at a time, each beginning as the previous ends. Under a second reading, these programs are co-initiated by the same signaling event and run concurrently from the outset, and what changes with time is not which program is active but which one predominates. The evidence assembled here favors the latter. IFN-γ induces checkpoint ligands and antigen-processing machinery in the same cells within the same early induction window through the same IRF1-dependent node (Section 3) (11), and in the CD4^+^ compartment the same IFN-γ-dependent response that produced initial tumor control also drove the T-cell attrition that left animals unable to mount secondary immunity (Section 6.2) (21). What is genuinely time-dependent is the relative weight of these co-active arms: a checkpoint ligand transcribed within hours restrains effectors only once PD-1^+^ cells have accumulated, so induction and functional consequence are necessarily separated in time; suppressive circuits stabilize only under sustained exposure (4); and selection can enrich cells that no longer transduce the signal only after repeated rounds of immune pressure (5).

Therefore, we use the IFN-γ Response Cycle to denote a temporal shift in the relative contribution of simultaneously initiated programs, not a succession of mutually exclusive biological states. The node designations used in Parts II–IV (execution, 1→2; the turn, 2→3; adaptation, 3→4) accordingly mark where each program’s contribution predominates, not intervals during which the others are absent. The loop is closed at the level of causal dependency between arms, in that productive signaling generates the conditions for its own restraint and for the enrichment of cells that escape it; it does not imply that an individual tumor oscillates between IFN-γ-sensitive and IFN-γ-resistant states, since genetic loss of pathway integrity is in general not reversible. The evidentiary basis for this claim of closure is set out below. Thus defined, the cycle is a proposed organizing interpretation of established mechanisms rather than an experimentally demonstrated temporal sequence, and we mark this distinction where it matters throughout.

Two features of the evidence base bear on how strongly this framework can be stated, and we set them out here rather than in the Limitations section. The first is provenance. The causal claims that give the cycle its structure rest almost entirely on murine work, whereas the human evidence supporting it is predominantly observational. The loss- and gain-of-function experiments establishing that IFN-γ signaling is required for a downstream outcome were performed in transplantable mouse tumors, most often melanoma: that IFNGR deletion restores the durable immunity otherwise lost to IFN-γ-dependent CD4^+^ attrition (21); that IFN-γ-dependent deletion of activated tumor-specific clones limits combination checkpoint blockade (22); that CD8^+^-derived IFN-γ enriches IFN-γ-signaling-mutant cells within a heterogeneous tumor (5); that STAT1 is required for the multigenic resistance program established by prolonged interferon exposure (23); and that sustained low-level IFN-γ promotes tumor growth reversibly, since checkpoint-ligand blockade abrogates it (4). Human data enter the argument in three weaker forms. Mechanistic work in patient-derived cell lines establishes molecular causation but not the behavior of a tumor within a host (11, 24). Genomic analyses of relapsed tumors recover loss-of-function lesions in JAK1, JAK2, and B2M from patients progressing on checkpoint blockade, but the selective history that produced them is reconstructed retrospectively rather than observed (6, 7). Correlative cohort studies relate IFN-γ activity to outcome without perturbing it (14, 15). The single prospective human intervention that speaks directly to the cycle is a phase 1 study in which IFN-γ administered with nivolumab raised monocyte PD-L1 in proportion to dose and duration, a direct human observation of the feedback arm, though in a small cohort and measured in blood rather than tumor (17).

This asymmetry matters for reasons specific to the claims made here. The cycle, as drawn in **Figure 1**, asserts the third, and we are not aware of a study that has demonstrated the closure of this complete circuit within a single experimental system. What exists is a set of separately established causal links supported across different experimental systems and, in some cases, different species. In the forward direction, IFN-γ signaling induces checkpoint-ligand expression through a JAK/STAT/IRF1 pathway (11), induces IDO1 through IFN-γ-responsive regulatory elements involving IRF1 and STAT1 (25), promotes IFN-γ-dependent deletion of activated tumor-specific T cells, and reshapes the clonotypic repertoire (22), and, in heterogeneous tumors, can select for the outgrowth of IFN-γ-insensitive clones (5). In the return direction, PD-1 blockade increases intratumoral IFN-γ and IFN-γ-inducible chemokines in a manner implicating host IFN-γ receptor signaling (26), and PD-L blockade suppresses the tumor-promoting effects of sustained low-level IFN-γ (4). The nearest single-system approximation to closure is the clonal-selection experiment, in which CD8^+^ T-cell-derived IFN-γ was shown to drive selection for IFN-γ-insensitive tumor-cell clones (5); however, the effect of this selected clonal composition on subsequent intratumoral IFN-γ output was not reported. When we describe the cycle as closed, we mean that these separately demonstrated links compose into a coherent circuit, not that the complete circuit has been observed to close within any one experimental system. Demonstrating closure would require serial measurement of IFN-γ output, checkpoint restraint, and clonal composition in the same tumors over time, together with perturbation of the relevant signaling axis at defined points; we identify this as the framework’s principal empirical gap.

Transplantable mouse tumors compress into weeks a selective process that in patients unfolds over months to years, and they typically begin from clonal or oligoclonal populations rather than from the established heterogeneity on which selection is proposed to act, a variable that the clonal-cooperation experiments themselves show to be decisive (5). System dependence is moreover documented within this literature: IFN-γ-driven metabolic restriction curbed growth in melanoma but not colon carcinoma (18), and single-cell analyses found the pathway’s prognostic correlation running in opposite directions across tumor types (15). We therefore state each mechanistic claim at the level at which it was demonstrated and identify the system in which it was obtained throughout Parts II–V, so that murine and human observations are not read interchangeably.

### 2.2 The molecular grammar: IFNGR–JAK–STAT1–IRF1 within the interferon network

IFN-γ engages a receptor–kinase–transcription-factor module, IFNGR1/2, JAK1/JAK2, STAT1 with STAT2/STAT3, and IRF1, that determines which genes it activates (11). Rather than a linear relay to a fixed output, this module behaves as a decoding system whose activated genes depend on which cell reads the signal and how it integrates with the wider interferon network; therefore, a conserved architecture can yield divergent programs.

The clearest evidence that one architecture serves opposing ends sits at IRF1. In melanoma cells, this axis was mapped as the dominant route to PD-L1, with IRF1 binding the PD-L1 promoter and PD-L2 co-regulated by IRF1 and STAT3 (11). The transcription factor that licenses antigen presentation and effector programs thus also drives checkpoint-ligand induction, placing IRF1 at a bifurcation where productive activation and negative feedback originate. This co-induction is directly observable rather than inferred: in the same melanoma lines, transcriptional profiling performed after three hours of IFN-γ exposure captured PD-L1 and PD-L2 alongside a second module of antigen-processing and presentation genes, including TAP1, TAP2, and PSMB8/PSMB9/PSMB10, of which TAP1 and PSMB9 are transcribed from a shared bidirectional promoter driven by the same STAT1–IRF1 axis (11, 12). Convergence of both arms on one node within a single early induction window is an argument for concurrent initiation; the ordering proposed in this review therefore refers to the subsequent divergence in how long each arm persists and how much it contributes, not to the order in which the arms are switched on (Section 2). Consistent with this, pre-treatment nuclear IRF1, the capability to mount the program rather than the checkpoint endpoint, tracked anti-PD-1 benefit more closely than PD-L1 in a small retrospective cohort (14).

This logic extends beyond that of IFN-γ alone. Type I and type II interferon programs, together with IL-15, converge on a shared immune-responsive state; a single readout, GBP1, expressed largely in macrophages and correlated with T cell-recruiting chemokines, appeared to capture this coordinated activation and to track checkpoint response (27). At single-cell resolution, the same pathway resolves into heterogeneous outputs: across tumor types, its prognostic correlation ran in both directions, and within tumors, it variably raised immunogenicity while restraining proliferation, with much of its regulatory weight in the stromal and immune compartments (15).

Overall, these observations support viewing the IFNGR–JAK–STAT1–IRF1 axis not as a fixed cascade but as a tunable backbone whose setting, by recipient cell, network context, and signal intensity, selects among antigen presentation, immune communication, checkpoint induction, and the resistance programs examined later (**Figure 2**). The first of these outputs, the execution of tumor killing, is where the productive arc of the cycle begins.

**Figure 2:**
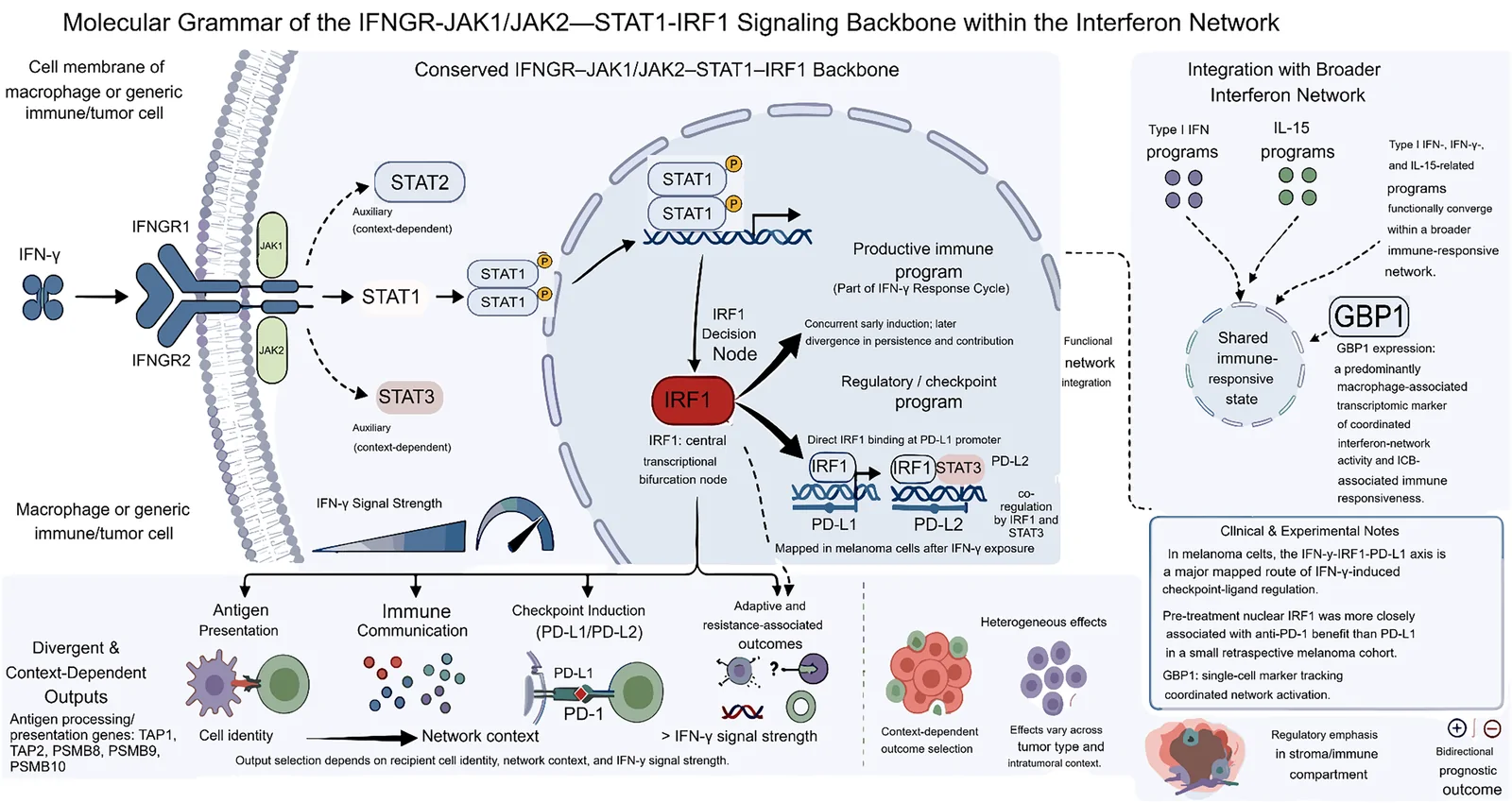
Molecular grammar of the IFNGR–JAK1/JAK2–STAT1–IRF1 signaling backbone within the interferon network. IFN-γ signaling through IFNGR1/2, JAK1/JAK2, STAT1, and IRF1 forms a conserved transcriptional module that integrates with broader interferon programs to generate context-dependent outputs. IRF1 acts as a bifurcation node linking antigen-presentation and immune activation programs with checkpoint induction (PD-L1/PD-L2), while GBP1 captures coordinated interferon-network activation within immune-responsive states. Cell identity, network context, and signal intensity determine the balance between productive immune programs, regulatory responses, and heterogeneous tumor-associated outcomes.

## 3 Part II. execution (node 1→2): how IFN-γ carries out tumor killing

### 3.1 Tumor-cell-intrinsic death programs

#### 3.1.1 Cytostasis, senescence, and apoptosis

Before IFN-γ recruits or amplifies immune killing, it acts directly on the tumor cell, and its earliest tumor-intrinsic outputs form a graded continuum of restraint rather than discrete events: weaker signaling slows the cycle, stronger or sustained signaling imposes a stable senescence-like arrest, and at the extreme, the same pathway commits the cell to death. Senescence is an active, required arm of control, exposed when its machinery is lost: in T-cell-sensitive tumors, deleting *Cdkn2a* or *Cdkn1a* abolishes both natural and checkpoint-driven control, while melanomas progressing rapidly under immune checkpoint blockade (ICB) carry losses of these genes and gains of senescence inhibitors (28). Arrest contains cells that escape outright killing, distinct from elimination in that the cell persists rather than disappears. Apoptosis extends the same axis to its terminus, IFN-γ raises phosphatidylserine and calreticulin on sensitive melanoma lines but not on a resistant one (29).

Whether a program reaches death depends on the competing survival inputs. In lung adenocarcinoma, IFN-γ engages JAK2–STAT1, carrying the antiproliferative effect, alongside PI3K–AKT, which supports survival and PD-L1; PI3K inhibition lowers PD-L1 and deepens arrest, and PD-L1 in IFN-γ-high tumors appeared to mark sensitivity, not mere shielding (30). Responsiveness thus spans a spectrum, from cells IFN-γ arrests or kills to states that read the signal yet do neither, and apoptosis-insensitive vaccine cells were even associated with better outcome (29), a context-bound result that still attests to IFN-γ-resistant states. These survivors—cells that resist arrest or death by losing senescence machinery or sustaining PI3K–AKT survival—are the population on which later resistance is built, so execution and selection are linked from the first encounter. Other IFN-γ killing arms operate in parallel and are considered next.

#### 3.1.2 Ferroptotic and metabolic death

Execution does not end with cell cycle arrest and apoptosis. IFN-γ also drives ferroptosis, an iron-dependent death executed by lipid peroxidation rather than caspases, and broader metabolic rewiring, and converging evidence frames these as effector arms of killing, not incidental accompaniments of immune activation. The ferroptotic arm has a defined mechanism. IFN-γ released by immunotherapy-activated CD8^+^ T cells downregulated SLC3A2 and SLC7A11, the two subunits of the glutamate–cystine antiporter system xc^-^, impaired tumor-cell cystine uptake, and consequently promoted lipid peroxidation and ferroptosis (31). In preclinical mouse models, depletion of cystine and cysteine using cyst(e)inase synergized with checkpoint blockade to enhance T cell-mediated antitumor immunity and induce tumor-cell ferroptosis (31). In human melanoma datasets, system xc^-^ expression was negatively associated with effector CD8^+^ T-cell and IFN-γ signatures, while lower SLC3A2 expression was associated with clinical benefit during nivolumab therapy and with patient outcome (31). Thus, the mechanism demonstrated in this study links IFN-γ signaling to ferroptotic lipid peroxidation through suppression of cystine uptake and consequent disruption of the cysteine/GSH-dependent antioxidant system. The contribution of ferroptosis to checkpoint efficacy is supported from both directions. Its suppression confers resistance: in TNBC, HMGB3 dampened STAT1–IRF1 signaling while raising the ferroptosis brakes SLC7A11, GPX4, and SLC3A2, and the resulting drop in IFN-γ-driven ferroptosis conferred anti-PD-1 resistance *in vivo*; HMGB3 expression also correlated with immunotherapy outcome in patients (32). Its enhancement improves response: in melanoma, an ATF3-induced miR-21-3p targeted TXNRD1 to amplify lipid ROS and ferroptotic sensitivity, and nanoparticle-delivered miR-21-3p synergized with anti-PD-1 (33).

Metabolic rewiring follows a parallel logic, though characterized in separate systems. In a murine hepatoma line, IFN-γ increased glycolytic flux and lactate production while suppressing mitochondrial function in a nitric oxide (NO)- and ROS-dependent manner, stabilized HIF-1α, and restrained growth rather than causing death (18). This program was heterogeneous: CT26 and B16F10 cells produced neither NO nor lactate, HIF-1α stabilization curbed growth in B16F10 but not CT26, yet exogenous lactate suppressed both, marking metabolic stress as a determinant of tumor-cell fitness engageable even where the NO arm is absent (18). Whether the metabolic and ferroptotic arms are mechanistically continuous remains open: their apparent point of contact is oxidative stress, but the ferroptotic arm proceeds through suppression of system xc^-^ and the cysteine/GSH axis rather than through NO (31), and NO can itself be anti-ferroptotic, since iNOS-derived NO• suppressed pro-ferroptotic 15-HpETE-PE formation in M1 macrophages (34). Current evidence therefore supports treating IFN-γ-driven metabolic rewiring and ferroptosis as parallel, context-dependent arms rather than steps of a single pathway.

Each arm can also be disabled using the mechanisms shown separately and at different levels: HMGB3 raises the ferroptosis brakes while dampening STAT1–IRF1 input (32), whereas the CT26 and B16F10 lines were constitutively unresponsive in the NO and lactate arms rather than having lost a previously present capacity (18). We regard these as potential non-adaptive forms of resistance; however, neither study demonstrated a transition of individual tumor cells from IFN-γ-sensitive to refractory states under sustained IFN-γ exposure.

### 3.2 Antigen presentation: the pivotal antitumor node

Among the tumor-intrinsic programs, antigen presentation is the most consequential, as it converts a cytokine signal into a target that the immune system can recognize. Where cytostasis and the death pathways constrain the cell, MHC-restricted presentation licenses its recognition, making this the node at which productive immunity is organized, rather than one more downstream effect of IFN-γ. The two presentation axes are not equivalent. MHC-I is near-ubiquitous, the dominant CD8^+^ substrate, and tumors carrying IFN-γ pathway defects but retaining high MHC-I expression remained responsive to checkpoint blockade in the models examined (35). MHC-II is more selective: across melanoma lines, it is bimodal and tumor-autonomous, IFN-γ-inducible in only a subset (36), and its function depends on processing enzymes such as GILT, whose high expression in tumor cells tracks better survival under checkpoint blockade (37).

Tumor-cell MHC-II behaves as an active biological state rather than a passive marker. In melanoma, it tracks response, survival, and CD4^+^/CD8^+^ infiltration (36), and in pancreatic cancer, it proved functional rather than shielding tumors via LAG-3, as earlier melanoma work had suggested; tumoral MHC-II contributed to CD4^+^/CD8+ cytotoxicity and presented HLA-II neoantigens (38). In head and neck cancer, an IFN-γ-driven malignant IFN/MHC-II program also marks responsive tumors (39). This suggests that MHC-II competence is not merely a biomarker but a mediator that shapes the magnitude and quality of the downstream response, including CD4^+^ helper cells, across the three tumor types.

This dependency carries a built-in liability. If the response is gated by antigen presentation, the machinery that generates benefit is also the tumor’s most rewarding target under immune pressure, which is why loss of MHC presentation later recurs as a dominant route of acquired resistance, the adaptation phase reading out at the cycle’s most pivotal node. Yet this node’s output is set not only inside the tumor cell but also by the immune populations producing and receiving IFN-γ, the cellular execution network we discuss next.

### 3.3 The cellular execution network: one signal, many decoders

#### 3.3.1 CD8^+^ cytotoxic effectors, autocrine/paracrine reinforcement

If the tumor cell is one reader of IFN-γ, the CD8^+^ T cell is another, peculiar in that it both produces the cytokine and responds to it. Within this compartment, IFN-γ acts less as a weapon aimed outward than as a self-reinforcing signal sustaining effector identity, the first case of a broader principle: the cell decoding IFN-γ, not the signal alone, sets the output. Two findings make this concrete. In mouse melanoma, CD8^+^ T cells lacking both PD-1 and LAG-3, but not either alone, cleared tumors and acquired an effector-like, interferon-responsive program with broad TCR clonality, requiring autocrine, cell-intrinsic IFN-γ signaling (40); the paired receptors otherwise drive exhaustion via TOX. In parallel, acetylation of T-cell-intrinsic p53 transactivated IFNG directly, establishing a p53–IFN-γ positive-feedback loop that amplified CD8 immunity independently of tumor p53 status (41).

The gain of this loop is set by two factors. Its durability tracks mitochondrial fitness: CD38-coupled mitochondrial dysfunction marks exhausted, ICB-resistant CD8^+^ T cells in lung cancer, and restoring that metabolism with combined PD-L1 blockade and EGCG revived IFN-γ output and survival (42). Its ignition is stochastic; across thousands of ex vivo assays, cytotoxicity followed a shifted-Poisson law traceable to rare “Spark” T cells whose paracrine IFN-γ feedback turns a chance activation into a macroscopic response (43). Collectively, the CD8^+^ compartment treats IFN-γ as an autocrine and paracrine amplifier whose gain is set by checkpoint and exhaustion state, mitochondrial fitness, and a few high-output cells; therefore, in the acute setting, “more IFN-γ” is less a dose than a property of the producing cell. This amplifier reading, however, captures only one arm of CD8^+^-intrinsic IFN-γ signaling. The same cytokine that reinforces effector identity can also constrain the CD8^+^ compartment when it acts on the cells that sustain it. Deleting the IFN-γ receptor specifically in mature CD8^+^ T cells improved tumor control in mouse melanoma, because chronic IFN-γ sensing preferentially depleted stem-like (TCF1^+^) CD8^+^ T cells—limiting their maintenance, proliferation, and clonal diversity—and IFNGR2 expression on circulating CD8^+^ cells correlated inversely with clonal size and with response to checkpoint blockade in patients with metastatic melanoma (44). Because CD8^+^ cells both produce and respond to IFN-γ, this constitutes a self-acting negative-feedback loop: the effector’s own product feeds back to erode the stem-like pool on which durable responses depend. The two arms are not contradictory but sequential in the same currency, autocrine IFN-γ can reinforce acute effector function while chronic sensing curtails the self-renewing reservoir behind it, so within a single lineage the cytokine both executes and, with persistence, limits itself. This intracellular execute-then-limit motif is the CD8^+^ counterpart of the tumor-cell feedback developed later (Sections 8–9) and, because it turns on a cell that is simultaneously the source and the target of the signal, is among the clearest cellular illustrations of the self-limiting turn at the center of the IFN-γ Response Cycle. However, whether this output ultimately helps or hinders depends on which other lymphocytes read it, beginning with the CD4^+^ compartment (**Figure 3**).

**Figure 3:**
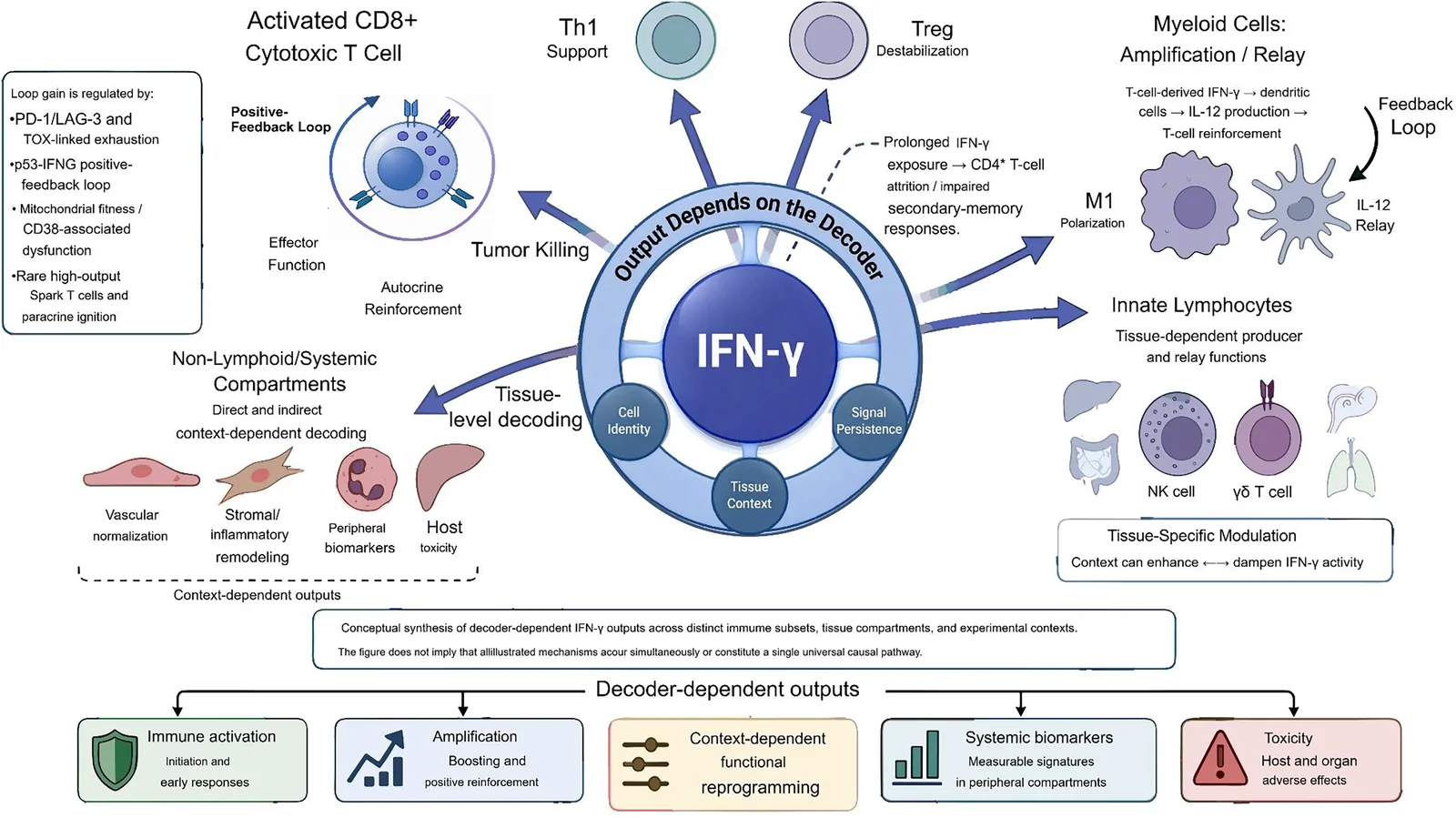
The cellular execution network of IFN-γ: one signal, many decoders. IFN-γ output is set by the decoder, cell identity, tissue context, and signal persistence, not by the signal alone. In CD8^+^ cytotoxic T cells, which both produce and sense IFN-γ, the cytokine drives two opposing arms: an acute autocrine positive-feedback loop reinforcing effector function and tumor killing (gain modulated by PD-1/LAG-3– and TOX-linked exhaustion, a p53–*IFNG* loop, mitochondrial fitness [CD38-associated dysfunction], and rare high-output “Spark” T cells), and a chronic negative-feedback arm in which sustained sensing depletes the stem-like (TCF1^+^) CD8^+^ pool, limiting durable responses. In CD4^+^ cells, IFN-γ supports Th1 activity and destabilizes Tregs, while prolonged exposure drives CD4^+^ attrition and impaired memory responses. Myeloid cells form an amplification/relay layer, with T-cell-derived IFN-γ licensing dendritic-cell IL-12 and M1 polarization. Innate lymphocytes (NK, γδ T cells) provide tissue-dependent producer and relay functions that context can enhance or dampen. In non-lymphoid and systemic compartments, IFN-γ yields context-dependent outputs including vascular normalization, stromal/inflammatory remodeling, peripheral biomarkers, and host toxicity.

#### 3.3.2 CD4^+^ helper and regulatory T cells: the conventional/regulatory axis

CD4^+^ T cells occupy a divided position in the IFN-γ network: the same cytokine that programs conventional helper cells toward antitumor support can dismantle the suppressive identity of regulatory cells. Sharing a receptor but sitting on opposite sides of the immune ledger, these subsets make the meaning of an IFN-γ signal depend less on the signal itself and more on which CD4^+^ cell receives it. Two observations show that this duality favors immunity. In gastric cancer transcriptomes, pembrolizumab responders were enriched for Th1 cells and IFN-γ-response genes, with Th1 cells inducing that response more efficiently than other populations and anchoring a predictive signature (45). In parallel, IFN-γ from destabilized intratumoral Tregs rendered neighboring regulatory cells functionally fragile, eroding their suppression, a transition the same study found necessary for responsiveness to PD-1 blockade (46). Helper programming and regulatory disarmament converge from opposite directions on the same output.

However, sustained signaling turns the same cytokine against the helper T cell arm. In several immunotherapy models, the CD4^+^ population, unlike CD8^+^ cells, failed to expand and instead underwent IFN-γ-dependent apoptosis, so that initially protective regimens left mice unable to mount secondary or memory responses; deletion of the IFN-γ receptor restored durability (21). Therefore, acute helper support and long-term maintenance of immunity appear to be in conflict with each other under the same signal. Read against the cycle, the CD4^+^ compartment marks the first inversion of recipient logic: one cytokine that strengthens immunity by arming helpers and disarming suppressors yet, with persistence, deletes the helpers on which durable control depends. Execution and self-limitation are not sequential but coincident, set by subset and exposure, a duality that widens once the signal reaches the myeloid cells that present the antigen and relay it onward (**Figure 3**).

#### 3.3.3 Myeloid decoders: macrophage repolarization and dendritic-cell licensing

If antigen presentation and the CD4^+^ axis set the terms of recognition, myeloid cells determine its reach. Macrophages and dendritic cells are the compartments in which IFN-γ is not merely received but relayed and converted through repolarization and cytokine relays into activation spreading across the microenvironment. In this way, they form the principal amplification layer of the cycle, extending immune pressure even where tumor cells are poorly antigenic or weakly IFN-γ-responsive. In macrophages, IFN-γ switches immunosuppressive, M2-polarized TAMs into M1-like immunostimulatory cells, restoring IL-12 and T-cell-supportive function while curbing trophic mediators and diverting monocytes from the TAM fate (47). This myeloid state, rather than tumor-cell-intrinsic sensitivity, can govern outcome, as evidenced by the failure of IFN-γ therapy against TAM-rich tumors despite equal intrinsic responsiveness, yet TAM depletion restored it (48). Reprogramming can go further: T-cell-derived IFN-γ, with damage signals from immunogenic cell death, drives NOS2^+^ tumoricidal macrophages that kill directly (49), while relieving tumor-derived MIF pushes macrophages toward M1 and augments checkpoint-driven CD8^+^ IFN-γ (50).

Two further observations turn activation into a circuit. Macrophages not only receive IFN-γ but can also produce it: under the epigenetic reader SP140, TAMs repressed STAT1 yet secreted IFN-γ, IL-12, and CXCL10 and adopted an antitumor phenotype, with high SP140 tracking checkpoint-blockade response across several cancers (51). Dendritic cells close the loop: a tumor-infiltrating DC subset, sensing T-cell-derived IFN-γ, produced the IL-12 that in turn re-stimulated those T cells, a reciprocal exchange that proved necessary for anti-PD-1 efficacy and could be amplified through non-canonical NF-κB (52). Across these settings, the logic converges: myeloid cells act as biological amplifiers, converting a T-cell-delivered IFN-γ signal into a self-reinforcing network of M1 macrophages and IL-12-licensed dendritic cells that sustains, and may be required for, durable checkpoint responses. Because much of this killing and licensing proceeds independently of tumor-cell IFN-γ sensitivity, myeloid amplification can raise immune pressure where the tumor itself has gone quiet. The same relay capacity extends to innate lymphocytes, the next sources and recipients of the signal (**Figure 3**).

#### 3.3.4 Innate lymphocytes: NK, NKT, and γδ T cells across tissue niches

Beyond αβ T cells, innate lymphocytes also produce and respond to IFN-γ. What distinguishes this compartment is not a separate program but its sensitivity to tissue context: the same population can amplify or stall the response depending on the organ in which it sits. NK cells with tissue-resident traits illustrate the relay role. Within the IFN-γ–IL-12 axis, CD49a^+^ NK cells produced CCL5, recruiting cDC1 and expanding dendritic-cell–CD8^+^ contacts; this population was required for intratumoral IL-12 and for PD-1-blockade efficacy in mouse models, and supplying CCL5 rescued tumors deficient in such cells (53). Here, innate cells initiate rather than kill, converting an IFN-γ-inducing therapy into adaptive, DC-organized immunity.

The γδ compartment shows how sharply the tissue conditions this output. Vδ1^+^ γδ T cells, a high-IFN-γ population at baseline, were depleted in mismatch-repair-deficient colorectal liver metastases, and their IL-18 stimulus was sequestered by tumor-derived IL-18-binding protein; intratumoral IL-18 tracked checkpoint benefit, and IL18BP blockade restored IFN-γ-driven killing *in vitro* (54). Because innate lymphocytes carry IFN-γ activity to sites beyond direct T cell recognition, the organ in which they reside can determine whether the pressure is amplified or muted. This may contribute to the reduced checkpoint benefit observed in patients with liver metastases, although the mechanism established for that clinical association is hepatic siphoning and macrophage-mediated deletion of activated CD8^+^ T cells rather than loss of γδ output (55), and the two have not been examined together. This dependence of a single lineage’s output on its anatomical setting recurs across the compartments considered below and is examined directly, together with its clinical correlation in hepatic metastasis, in Section 6.6 (**Figure 3**).

#### 3.3.5 Non-lymphoid and systemic decoders: endothelium, stroma, granulocytes, and host toxicity

IFN-γ also acts on non-immune cells. Endothelium, stroma, and distant tissues respond to it, so its effects extend beyond the tumor to the whole organism. Endothelial and stromal responses bracket the divergence this produces. In a lung-adenocarcinoma model, IFN-γ generated during PD-L1 blockade suppressed the ANGPT2–Tie2 program in endothelial cells through STAT1 binding at the ANGPT2 and TEK promoters and AKT–FOXO1-mediated repression of ANGPT2, normalizing the tumor vasculature and contributing to efficacy (56). In a melanoma model, the same upstream signal turned destructive: T-cell-derived IFN-γ drove macrophage TNF-α that converted fibroblasts into inflammatory CAFs capable of promoting progression (57). Vascular normalization and stromal remodeling emerge from one cytokine read by two compartments.

Peripheral-blood correlates of interferon activity, in which markers of benefit and of toxicity overlap, are considered as biomarkers in Section 17. Two observations should be distinguished because they are easily conflated. First, antitumor activity and host toxicity may arise from overlapping IFN-γ signaling, but the available evidence does not establish that they are generated by the same cytokine pool or cellular source. Cardiac injury in a murine model involved IFN-γ–STAT1 signaling in macrophages (58), illustrating that the same canonical pathway used for antitumor immune activation can also mediate tissue injury. Clinically, irAEs are consistently associated with treatment benefit: a meta-analysis of 52 studies involving 9,156 patients found associations with objective response (OR 3.91), PFS (HR 0.54), and OS (HR 0.51), although high-grade immune-related adverse events (irAEs) were not associated with improved outcomes and the association was stronger for PD-L1 than CTLA-4 blockade (59). A secondary analysis of a randomized pembrolizumab-versus-placebo trial likewise found longer recurrence-free survival among patients who developed irAEs (60). In an NSCLC cohort, circulating IFN-γ and eosinophils were associated with both irAEs and more favorable outcomes, but these findings are observational and do not establish a shared mechanism (61).

Second, the anatomical source of IFN-γ responsible for tissue toxicity remains unresolved. It could derive from systemic cytokine exposure or from effector cells producing IFN-γ locally after trafficking to affected tissues; current clinical measurements cannot distinguish these possibilities. Supporting this distinction, systemic recombinant IFN-γ given with nivolumab caused dose-related constitutional and laboratory toxicities, including transaminase elevation and grade 3–4 lymphopenia, but only one of 26 patients developed an immune-related adverse event, and none of grade 3 or higher (17). The investigators therefore proposed, provisionally, that IFN-γ exposure might not promote classical irAEs. Definitive source attribution would require tissue- and cell-type-specific manipulation of IFN-γ signaling or production. Such approaches are established in mice, where restricting IFN-γ receptor expression to defined cell types has been used to assign the target compartment of IFN-γ action (62), but no equivalent method is available for defining the origin of immunotherapy toxicity in patients. Accordingly, efficacy–toxicity coupling should be interpreted as evidence of potentially overlapping signaling biology, not as proof that the same IFN-γ source produces both outcomes. Read across these compartments, the lesson of this section is that IFN-γ is not a tumor-confined effector but a widely decoded signal whose outputs, vascular normalization, stromal remodeling, systemic biomarkers, and host injury, emerge from one signaling cascade read in different tissues. The reach of antitumor immunity and the risk of collateral injury are therefore properties of the same molecule, though, as set out above, whether they arise from the same pool of it remains undetermined (**Figure 3**).

#### 3.3.6 Tumor-type and organ-site heterogeneity: the limits of generalization

The preceding subsections are organized by cell type, which risks implying that each lineage has a characteristic IFN-γ response. The available evidence does not support this premise. Where related populations have been examined in different settings, their functional responses to inflammatory cues and the downstream consequences of IFN-γ signaling have differed by tumor type, anatomical site, and stromal context, and in several cases, the difference is not one of magnitude but of mechanism.

The γδ compartment provides a clear within-lineage contrast. The same Vδ1^+^ depletion and IL-18BP constraint that limits IFN-γ in MMR-deficient colorectal liver metastases (54), reverses in a distinct intestinal setting, where γδ T cells expanded to produce IFN-γ, upregulated CXCR3, migrated to distant tumors, and synergized with PD-L1 blockade (63). The endothelium shows a comparable divergence at the level of mechanism. In a murine Lewis lung carcinoma model, IFN-γ signaling during PD-L1 blockade suppressed the ANGPT2–Tie2 program and promoted tumor vascular normalization, contributing to antitumor efficacy (56). In mice engineered to restrict IFN-γ receptor expression to defined cell types, however, endothelial responsiveness was necessary and sufficient for IFN-γ-induced regression of large, established tumors, acting through vascular regression, arrest of blood flow, and subsequent ischemic collapse (62). Vascular normalization and extensive vascular regression therefore represent distinct vascular outcomes that can each contribute to tumor control, and their predominance cannot be inferred from cell identity alone. The same study also examined fibroblasts: responsiveness to IFN-γ by fibroblasts, myeloid cells, or T cells was not sufficient for tumor regression (62), whereas in melanoma, T-cell-derived IFN-γ induced macrophage TNF-α, which in turn converted fibroblasts into inflammatory CAFs that promoted tumor progression (57). Stromal decoding is therefore neither uniformly required across stromal compartments for tumor control nor uniformly protumor.

Heterogeneity of this kind can also be measured at the level of the signal itself, including within a single histology. In mucosal melanoma, IFN-γ signature levels varied by anatomical region, and immune-infiltrated tumors that were unresponsive to checkpoint inhibition showed higher fibroblast, macrophage, and TGF-β-associated signals, suggesting that IFN-γ-associated immune activity may have different functional consequences in distinct stromal states (64). Findings obtained in one tumor type or anatomical site should therefore be generalized to other solid tumors with caution, particularly when the relevant stromal or organ-specific context differs. Accordingly, we indicate the setting of each observation throughout this review rather than presenting mechanisms as lineage-general.

Hepatic metastasis provides a clear clinical example of organ-site heterogeneity. In patients with melanoma or non-small-cell lung cancer treated with pembrolizumab, liver metastasis was associated with reduced response and shorter progression-free survival; in the melanoma cohort, objective response was 30.6% versus 56.3% and median progression-free survival was 5.1 versus 20.1 months, with the association confirmed in an independent validation cohort (65). Liver metastasis was also associated with reduced CD8^+^ T-cell density at the invasive tumor margin (65). The established preclinical mechanism is not IFN-γ-specific: liver metastases promote FasL-dependent deletion of activated CD8^+^ T cells by hepatic macrophages, causing systemic T-cell loss and reduced efficacy, reversible by liver-directed radiotherapy in mice (55). The γδ/IL-18BP axis (54) operates in the same compartment, but the two have not been examined together, so we do not propose IFN-γ dysregulation as the explanation; rather, the liver illustrates how distinct organ-specific mechanisms can limit antitumor immunity within one anatomical site.

### 3.4 The spatial logic of execution

Execution depends not only on which cells respond to IFN-γ but also on where they respond. The same activated populations can succeed or fail by their arrangement, and a recurring lesson is that spatial organization, not bulk cytokine or cell abundance, governs whether an IFN-γ-driven response becomes tumor elimination. This logic runs from multicellular clusters to receptor routing inside one cell. The clearest case is the intratumoral triad. In mouse adoptive therapy and checkpoint blockade models, responses were dictated by the spatial positioning of CD8^+^ and CD4^+^ T cells, not their numbers: a CD4^+^ cell, a CD8^+^ cell, and a shared dendritic cell had to cluster during the effector phase to license CD8^+^ cytotoxicity (66). When disrupted, tumors progressed despite equal T-cell numbers, and triads were associated with response in pleural mesothelioma.

The same primacy of arrangement appears at a more refined scale. In metastatic melanoma, a spatial PD-1/PD-L1 interaction score and indoleamine 2,3-dioxygenase 1 (IDO1)/HLA-DR co-expression, colocalized IFN-γ-inducible markers, predicted anti-PD-1 response and survival, whereas the individual markers, including PD-L1, did not (67). Moreover, ARF6-dependent recycling sets the surface density of the IFN-γ receptor on melanoma cells; loss of Arf6 confers checkpoint resistance in mice and correlates with inferior post-ICB survival in patients (68). Across these scales, spatial organization determines whether IFN-γ signaling culminates in tumor elimination: IFN-γ culminates in elimination only where its signals and the cells that read them are concentrated, colocalized, and correctly routed, and disorganization stalls even when activated cells are abundant. Adding space to the intensity, recipient, and kinetics sets up the synthesis of these determinants, intensity, recipient cell, kinetics, and spatial organization, into the cycle’s systems-level logic, where the same signal that executes begins to limit itself.

## 4 Part III. The turn (node 2→3): the productive signal becomes self-limiting

### 4.1 Adaptive immune resistance: IFN-γ-driven checkpoint induction

The same signal that executes also sows its constraints. Adaptive resistance is best understood as the late-dominant arm of productive IFN-γ signaling rather than as a separate protumoral program: its transcriptional components are induced together with the productive ones (Section 3), but their net restraining effect emerges only because immune engagement has been successful. The transition from execution to restraint is therefore a kinetic event, a turn rather than a switch, in which amplification and limitation are concurrent components of one engagement whose balance tips over time. The productive arc is visible in the functioning of checkpoint blockade. In a murine adoptive-transfer model, anti-PD-1 raised tumor IFN-γ and the IFN-γ-inducible chemokine CXCL10, and increased T-cell accumulation and regression; experiments in IFNGR-deficient hosts placed IFN-γ as the nexus controlling this trafficking (26). Here, IFN-γ does exactly what the execution phase requires: it recruits and concentrates effectors at the tumor site.

Yet, this engagement spawns restraint. CD8^+^ activation unleashed by PD-1 blockade can trigger a tumor-intrinsic PD-L1/NLRP3 inflammasome cascade that recruits granulocytic MDSCs and dampens the response; NLRP3 inhibition restores efficacy (69). IFN-γ also directly induces checkpoint molecules on tumor cells: in melanoma, it drives CTLA-4 transcription via STAT1 binding to a gamma-activated sequence and induces an IFN-γ response signature including CTLA-4 that tracks a durable anti-CTLA-4 response (24). Nor does the inhibitory signal need to stay membrane-bound: metastatic melanomas release exosomes carrying surface PD-L1, and IFN-γ stimulation raises the amount of PD-L1 on those vesicles, which suppresses CD8^+^ T-cell function beyond the immunological synapse (70). The export machinery itself can be regulated independently of IFN-γ; in hepatocellular carcinoma, an ANXA2–CD63 axis stabilizes exosome secretion and exosomal PD-L1 (71). These circuits are not equivalent in how firmly IFN-γ is established as their initiating driver, and we distinguish three categories throughout Parts III and IV. Some are induced directly, with IFN-γ or STAT1 shown to be required or sufficient: CTLA-4 transcription in melanoma is driven by STAT1 occupancy at a gamma-activated sequence (24), and IFN-γ stimulation increases the PD-L1 carried on melanoma-derived exosomes, with circulating exosomal PD-L1 correlating with IFN-γ levels in patients (70). Others are IFN-γ-enabled but co-dependent, in that IFN-γ supplies a necessary component while a second signal provides the proximate trigger; the PD-L1/NLRP3 cascade is of this type, since IFN-γ induces the PD-L1 whose subsequent cross-linking, following CD8^+^ T-cell activation, initiates inflammasome signaling (69). A third category comprises mechanisms that accompany an IFN-γ-rich response without IFN-γ having been shown to initiate them, such as the ANXA2–CD63 axis that stabilizes exosome secretion and exosomal PD-L1 in hepatocellular carcinoma, which is regulated tumor-intrinsically and was not examined as an IFN-γ-responsive process (71). What unites all three is their association with immune engagement; only the first represents a directly demonstrated IFN-γ feedback arm. It follows, though untested directly, that the more effective and sustained the IFN-γ signal, the greater the opportunity for these self-limiting programs to engage (**Figure 4**). The productive response thus carries the seed of its own constraint, with effective immune engagement progressively giving rise to the mechanisms that limit its persistence.

**Figure 4:**
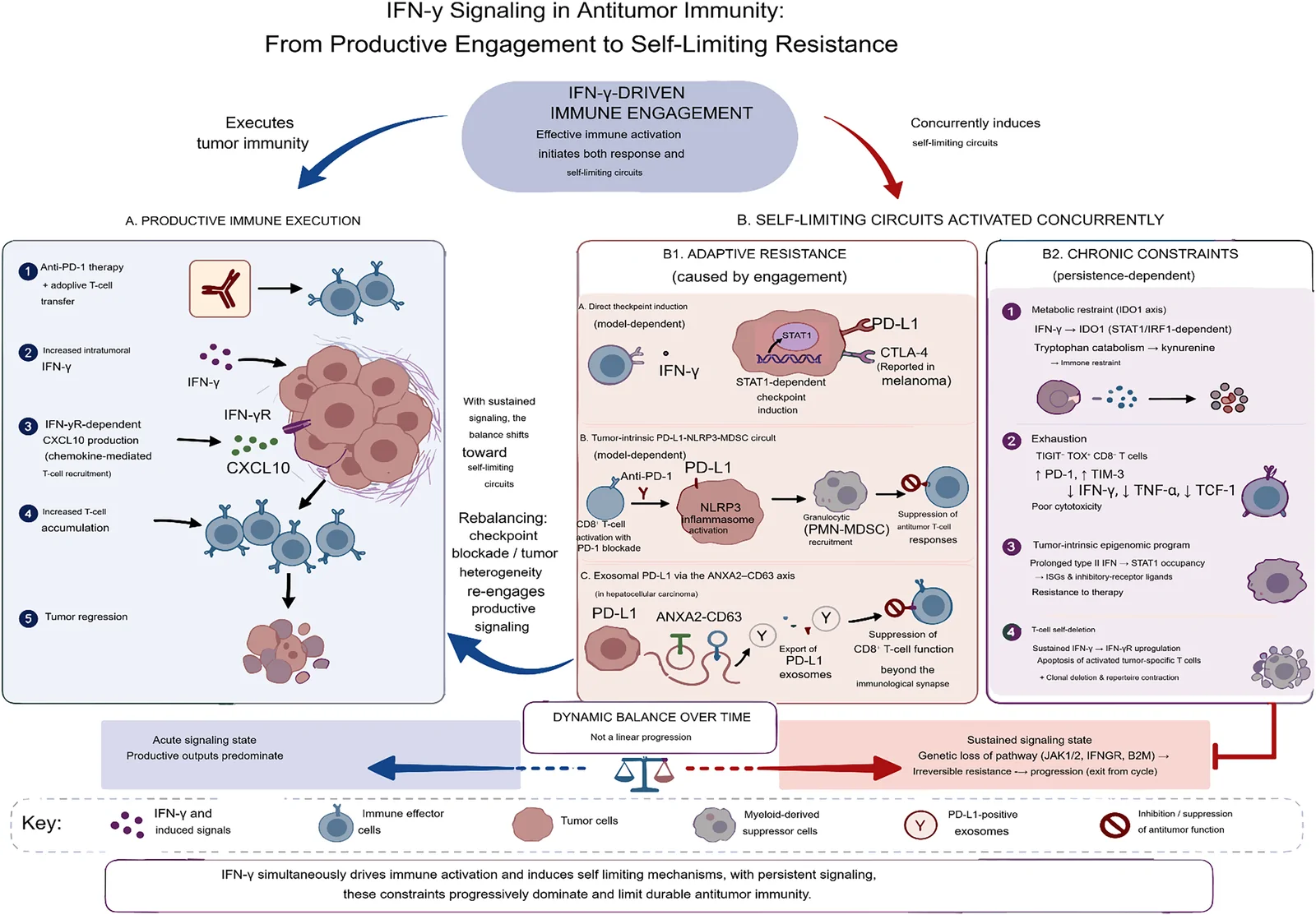
The transition of IFN-γ signaling from productive immune engagement to self-limiting resistance. **(A)** Productive immune execution: IFN-γ-driven activation promotes CXCL10-dependent T-cell recruitment, intratumoral accumulation, and tumor regression following effective immune engagement. **(B)** Self-limiting circuits activated in parallel: adaptive resistance emerges as a concurrent consequence of immune engagement, including (B1) checkpoint induction and PD-L1-associated suppressive circuits, and (B2) persistence-dependent chronic constraints involving metabolic restriction, exhaustion-associated states, tumor-intrinsic resistance programs, and T-cell deletion. The balance between productive outputs and self-limiting mechanisms shifts over time with sustained signaling, representing a dynamic constraint on durable antitumor immunity rather than a linear transition between discrete phases.

### 4.2 Chronic feedback: suppressive metabolism, exhaustion, and T-cell self-deletion

Adaptive resistance is initially reversible, but under sustained IFN-γ exposure it can stabilize into durable suppression. Brakes that are applied for a long time become structural. Metabolic restriction is the first circuit. IDO1-driven tryptophan catabolism behaves as a resistance mechanism to checkpoint blockade: IDO deletion or inhibition synergized with anti-CTLA-4 and anti-PD-1 to reject even poorly immunogenic, IDO1-non-expressing tumors in a T-cell-dependent manner, raising effector-to-Treg ratios (72). In IDO1-nonexpressing tumor models, the relevant IDO1 was host-derived, and IFN-γ dependence was not directly tested. The link to IFN-γ is established instead at the promoter: IFN-γ-inducible human IDO1 expression depends on coordinated IRF1- and STAT1-responsive regulatory elements (25), placing IDO1 among the direct transcriptional outputs of IFN-γ–STAT1/IRF1 signaling pathway. Consistent with this, IFN-γ stimulation of hepatocellular carcinoma cells increased IDO1, JAK1, and phosphorylated STAT1, enhanced RNA m6A methylation, and increased tryptophan metabolism toward kynurenine; the IDO1 inhibitor abrine attenuated these effects (73). Because these experiments used acute rather than sustained IFN-γ exposure, they support inducibility but do not establish how the circuit behaves under chronic signaling.

The constraints then broaden from metabolism to T cells and to a coordinated tumor program, although the two are not established on the same evidentiary footing. In extrahepatic cholangiocarcinoma, TIGIT marked TOX^+^CD8^+^ T cells with reduced IFN-γ, TNF-α, and TCF-1 and elevated PD-1 and TIM-3, an exhausted, poorly cytotoxic state linked to worse survival and partly reversed by anti-TIGIT (74). This is an association with an IFN-γ-rich, chronically stimulated microenvironment rather than a demonstrated IFN-γ-initiated circuit: TIGIT and TOX are induced by persistent antigen receptor signaling, and neither IFN-γ nor STAT1 was manipulated in that study. The tumor-intrinsic program, by contrast, is causally anchored. Prolonged type II interferon exposure altered the melanoma epigenome *in vivo*, with STAT1 occupancy associated with these changes and driving elevated expression of interferon-stimulated genes and of ligands for multiple T-cell inhibitory receptors; STAT1 knockout inhibited resistance to radiation plus anti-CTLA-4, and combined STAT1 and PD-L1 deletion improved response beyond either alone (23). Notably, while persistent IFN-γ signaling was sufficient to establish this program, both type I and type II interferons maintained it, so the two arms cannot be fully separated once the resistant state is set. At the far end lies physical attrition. Activated tumor-specific T cells upregulated the IFN-γ receptor and were apoptosis-prone, and combined anti-CTLA-4/anti-PD-1, especially at low tumor burden where per-cell IFN-γ peaked, deleted tumor-specific clones and contracted the repertoire (22). Taken together, these findings describe constraints that may differ in durability, and it is important to distinguish those for which IFN-γ is an established driver. Sustained IFN-γ signaling is a demonstrable driver of three: the metabolic step, through STAT1- and IRF1-dependent induction of IDO1 (25, 73); the transcriptional step, through epigenomic remodeling and a multigenic resistance program (23); and the deletional step, through IFN-γ-dependent apoptosis and deletion of activated tumor-specific T cells (22). Exhaustion, by contrast, is associated with chronic antigenic stimulation, but its relationship to IFN-γ signaling is not established by the cited study (74). We therefore treat the ordering of these constraints as a framework of increasing durability, from a potentially reversible metabolic constraint to deletion of responding clonotypes, rather than as a single causal cascade in which each step produces the next (**Figure 4**). Therapeutically, this distinction suggests that interventions targeting IFN-γ signaling should have their most direct effects on the IFN-γ-initiated steps.

## 5 Part IV. adaptation (node 3→4): the selectable landscape of resistance

### 5.1 Gating the signal down: making the tumor refractory

#### 5.1.1 Metabolic gating of IFN-γ responsiveness

A signaling pathway can be intact and still go unnoticed. Beyond the genetic lesions that disable IFN-γ signaling, the metabolic state of a cancer cell determines whether the pathway is competent to fire, so that metabolism controls responsiveness rather than serving only as an energy source. The same intact pathway can yield a productive response or none, depending on nutrient and biosynthetic states. **Table 1** assembles the mechanisms discussed in this and the next sections along a single axis of escalating permanence, from the reversible gating of a structurally intact signal to hard-wired genetic escape and plasticity-driven states that close the adaptation arc. Nutrient supply acts at both ends of the signal. On the receiving side, glutamine deprivation drives autophagy-dependent degradation of the IFN-γ receptor IFNGR1 in lung cancer, blunting the response and conferring anti-PD-1/PD-L1 resistance; restoring lysosomal control rescued both the receptor and the response (75). On the production side, targeting asparagine in gastric cancer enhances CD8^+^ T-cell infiltration and their IFN-γ and chemokine output, synergizing with anti-PD-L1 in a CD8-dependent manner (76).

**Table 1:** The selectable landscape of IFN-γ resistance, parallel mechanisms across the adaptation arc (Node 3→4) of the IFN-γ response cycle.

Mechanism	Reversibility	Cycle node	Evidence (key finding · system · caveat)	Ref.
A. Metabolic gating of IFN-γ responsiveness				
Glutamine deprivation → IFNGR1 loss *autophagy-dependent receptor degradation*	Reversible	3→4 Adaptation *reversible gate (reception)*	Glutamine restriction drove autophagy-dependent IFNGR1 degradation, blunted the IFN-γ response, and conferred anti-PD-1/PD-L1 resistance; restoring lysosomal control (V9302, chloroquine) rescued the receptor and response. *Lung cancer (lines, PDX, syngeneic).*	(75)
Asparagine targeting *producer-side; boosts effector IFN-γ*	Reversible	3→4 Adaptation *reversible gate (reception)*	Targeting asparagine-mediated CD8^+^ T-cell infiltration and their IFN-γ/CXCL9-10/GZMB output, synergizing with anti-PD-L1 in a CD8-dependent manner. *Gastric cancer models.*	(76)
Loss of bisecting N-glycosylation (MGAT3↓) *proteasomal IFNGRα destabilization*	Reversible	3→4 Adaptation *reversible gate (reception)*	Down-regulated MGAT3 destabilized IFNGRα via proteasomal degradation, producing intrinsic IFN-γ resistance, which could be reversed by restoring MGAT3 or all-trans retinoic acid. *Colorectal cancer.*	(77)
PFKFB3-high glycolytic state *broad immunosuppressive correlate*	Reversible	3→4 Adaptation *reversible gate (reception)*	PFKFB3 was elevated in CRC and in PD-L1-blockade-resistant patients with shorter survival; suppression reduced tumorigenesis and improved immunotherapy. *The associations were largely correlative and bioinformatic.*	(78)
B. Epigenetic & epitranscriptomic gating of the IFN-γ/antigen-presentation axis				
PRC2/EZH2 (H3K27me3) *suppresses tumor-specific MHC-II*	Reversible	3→4 Adaptation *reversible gate (transcription)*	PRC2/EZH2 hypermethylation suppressed tsMHC-II and its IFN-γ inducibility (likely via reduced CIITA accessibility); PRC2 inversely correlated with anti-PD-1 response, and EZH2 inhibition restored MHC-II expression. *Melanoma.*	(79)
DNA methylation (DNMT) *low-dose 5-azacytidine, clinical*	Reversible	3→4 Adaptation *reversible gate (transcription)*	In ICB-refractory disease, 5-azacytidine increased the IFN-γ signature and PD-L1 with more CD8^+^ and fewer Tregs, correlating with prolonged OS on rechallenge. *HNSCC; small Phase 1b (3/8 evaluable with >2-fold rise).*	(80)
HSP90 chaperone *ganetespib amplifies ISGs*	Reversible	3→4 Adaptation *reversible gate (transcription)*	HSP90 inhibition up-regulates interferon-response genes required for enhanced T-cell killing and potentiates anti-CTLA-4/anti-PD-1. *Melanoma (autologous T-cell killing; in vivo).*	(81)
METTL3/YTHDF2 (m6A) *epitranscriptomic checkpoint*	Reversible	3→4 Adaptation *reversible gate (transcription)*	METTL3 inhibition triggered a tumor-intrinsic interferon response; YTHDF2 loss prevented MHC-I degradation, with strengthened T-cell IFN-γ/GzmB and dampened exhaustion. *Melanoma, MC38 models.*	(82)
METTL3 (immune-side, m6A) *SCAP/cholesterol restrains CD8^+^*	Reversible	3→4 Adaptation *reversible gate (transcription)*	METTL3 restrains IFN-γ^+^ CD8^+^ infiltration via SCAP-driven cholesterol metabolism; its targeting synergized with anti-PD-1. *NAFLD-associated HCC.*	(83)
C. Oncogenic & environmental gating				
MYC → JAK2 repression *E-box in JAK2 promoter*	Reversible	3→4 Adaptation *baseline gate (upstream)*	MYC repressed JAK2 through a promoter E-box, lowering IFN-γ responsiveness; this was reversed by silencing MYC or MAX. *Melanoma.*	(84)
EGFR → SHP2 → STAT1 *premature STAT1 dephosphorylation*	Reversible	3→4 Adaptation *baseline gate (upstream)*	EGFR activation drove SHP2 to dephosphorylate STAT1, prematurely terminating the IFN-γ response and curbing chemokine/antigen presentation output; SHP2 inhibition restored sensitivity. *Lung and other cancer models.*	(85)
miR-1246 – AGO2 complex *stabilizes PTPN6, damps STAT1*	Reversible	3→4 Adaptation *baseline gate (upstream)*	An IFN-γ-induced miR-1246–AGO2 complex stabilized PTPN6 mRNA, dampening STAT1 phosphorylation and ISG/HLA output; reversible with BCI-137 or an antagomir. *Preclinical cancer models.*	(86)
Tumor-infiltrating microplastics *environmental; JAK–STAT–microbiota*	Reversible	3→4 Adaptation *baseline gate (upstream)*	Microplastics inhibited JAK–STAT, downregulated IFN-γ, CXCL9/11, B2m, and H2-K1, reduced T-cell infiltration, and induced microbiota dysbiosis, worsening resistance. *Colorectal cancer (animal models).*	(87)
D. Hard-wired genetic escape: loss of the IFN-γ pathway (JAK/B2M)				
IFN-γ pathway gene defects *anti-CTLA-4 non-responders*	Permanent	3→4 → Node 4 *fixed (selected) escape*	Genomic defects in IFN-γ pathway genes were enriched in anti-CTLA-4 non-responders, and IFNGR1 knockdown was sufficient to blunt tumor rejection in these patients. *Melanoma (patients; mice).*	(7)
JAK1/2 inactivating mutations *primary resistance*	Permanent	3→4 → Node 4 *fixed (selected) escape*	Loss-of-function JAK1/2 mutations abolished reactive PD-L1 induction and IFN-γ response, underlying primary anti-PD-1 resistance. *Melanoma and MMR-deficient colon cancer.*	(88)
Biallelic JAK1/2 loss; B2M truncation *acquired resistance at relapse*	Permanent	3→4 → Node 4 *fixed (selected) escape*	Relapsing lesions after initial regression carried biallelic JAK1/JAK2 loss (insensitivity to IFN-γ antiproliferation) or a truncating B2M mutation that collapsed surface MHC-I. *Melanoma (paired biopsies).*	(6)
JAK1 ≫ JAK2 dependency *non-equivalent kinases*	Permanent	3→4 → Node 4 *fixed (selected) escape*	JAK1 was the principal conduit for IFN-γ-driven MHC-I/II, PD-L1 and cytostatic responses, whereas JAK2 was largely dispensable; PTPN2 loss potentiated signaling. *Melanoma (mechanistic dissection).*	(89)
CDKN2A loss co-deleting JAK2 *structural susceptibility*	Permanent	3→4 → Node 4 *fixed (selected) escape*	Frequent CDKN2A chromosomal loss co-deleted JAK2, heightening susceptibility to IFN-γ resistance (odds ratio ≈ 223). *Melanoma and pan-cancer TCGA.*	(90)
B2M mutation/LOH *antigen-presentation collapse*	Permanent	3→4 → Node 4 *fixed (selected) escape*	B2M point mutations, deletions, or LOH were enriched ~3-fold in non-responders across anti-CTLA-4 and anti-PD-1, with biallelic loss only in non-responders and poorer survival. *Metastatic melanoma cohorts.*	(91)
Circumventing genetic resistance *innate/NK re-routing*	Permanent	3→4 → Node 4 *fixed (selected) escape*	JAK1/2- and B2M-knockout resistance was overcome by rerouting immunity through NK and innate effectors (intratumoral TLR9 agonist; CD122-biased IL-2). *The lesion is permanent but selective, not absolute.*	(92)
Maturation to HLA-I-negative lesions *genetic→epigenetic convergence*	Permanent	3→4 → Node 4 fixed (selected) escape; epigenetic consolidation	Acquired IFN-γ resistance, often initiated by JAK1/2 lesions detectable pre-treatment, matures into HLA-class-I-negative lesions with silenced, non-IFN-γ-inducible antigen-presentation genes. *Melanoma patient models, genetic core, epigenetic silencing.*	(93)
IFN-γ-induced dedifferentiation *neural-crest-like state*	Reversible	3→4 Adaptation *reversible phenotypic axis*	Prolonged IFN-γ treatment drove differentiated melanoma cells toward a neural-crest-like state via global chromatin remodeling, and the same signature was also associated with improved outcomes. *Melanoma (in vitro + patient correlates); directionality is context-dependent.*	(94)
E. The selectable landscape & clonal dynamics of escape				
*In vivo* CRISPR — PTPN2 *maps selectable determinants*	Context-dependent	3→4 Adaptation *ecological selection*	Screens recovered IFN-γ-pathway/antigen-presentation determinants of resistance while nominating PTPN2, whose loss enhances (not impairs) IFN-γ effects. *Transplantable melanoma; enumerates what could be selected.*	(95)
IFN-γ sensing is double-edged *NK/NKG2A · Qa-1b/HLA-E*	Context-dependent	3→4 Adaptation *ecological selection*	Loss of tumor IFN-γ signaling sensitized some models via NK and NKG2A (Qa-1b/HLA-E), and strong IFN signatures were associated with poor ICB response. *Pan-cancer screens: RCC and melanoma cohorts.*	(96)
CHD1 and MAP3K7 (TAK1) *tune, not abolish, the response*	Context-dependent	3→4 Adaptation *ecological selection*	Loss of CHD1 or MAP3K7 potentiated the IFN-γ transcriptional response, sensitizing cells to T cells, and reduced expression correlated with the ICB response. *Tumoroid–T-cell co-cultures; isogenic models.*	(97)
Clonal cooperation *WT-supplied PD-L1 shields mutants*	Context-dependent	3→4 Adaptation *ecological selection*	IFNGR2-/JAK1-deficient clones were better controlled in isolation (defective PD-L1) but expanded when admixed with wild-type cells whose PD-L1 shielded them — a selective value set by the clonal context. *B16.SIY melanoma.*	(5)
Neoantigen erosion + IFN-γ decline *explosive outgrowth*	Context-dependent	3→4 Adaptation *ecological selection*	Explosive tumor outgrowth coincided with reduced INDEL-derived neoantigens and diminished IFN-γ signaling, resulting in the simultaneous collapse of recognition and responsiveness. *Single colon cancer patient (Lynch syndrome); correlative.*	(98)
F. Induced phenotypic states: dedifferentiation, antigen remodeling, stem-like state (directionality context-dependent)				
MITF-low/AXL-high state *transcriptional MHC-I loss; TGFβ*	Reversible	3→4 → Node 4 fixed (selected) escape; epigenetic consolidation	A dedifferentiated MITF-low/AXL-high phenotype with transcriptional MHC-I down-regulation marked PD-1 progression and was driven by TGFβ. *Melanoma (baseline vs. progression biopsies).*	(99)
IDO1 → tryptophan depletion *ribosomal frameshifting; aberrant peptides*	Reversible	3→4 → Node 4 induced state; associated with improved recognition or outcome	IFN-γ-induced IDO1 tryptophan depletion provokes ribosomal frameshifting (‘W-bumps’) and surface presentation of aberrant trans-frame peptides — peptidome remodeling, not erasure. *Melanoma (ribosome profiling and immunopeptidomics).*	(100)
TCF4 regulatory network *mesenchymal-like, AP-low program*	Reversible	3→4 → Node 4 fixed (selected) escape; epigenetic consolidation	TCF4-centered network enforced a mesenchymal-like, antigen-presentation-low program enriched in early non-responder biopsies; TCF4 inhibition (BET) restored immunogenicity. *Melanoma (single-cell, spatial multi-omics).*	(101)
IFN-γ → stem-like conversion *BCAT1 mediator*	Reversible	3→4 → Node 4 fixed (selected) escape; epigenetic consolidation	IFN-γ directly converted non-stem cells to stem-like cells via BCAT1, conferring cytotoxic resistance and metastatic competence. BCAT1 blockade improved vaccination/ICB. *Breast cancer models: human ICB correlation.*	(16)
Transient cell-in-cell structures *STAT3/EGR-1; shelter & reseed*	Reversible	3→4 → Node 4 fixed (selected) escape; epigenetic consolidation	IFN-γ-activated T cells induce transient cell-in-cell structures (STAT3, EGR-1) that shield inner cells from killing and reseed tumors after T cells withdraw, resulting in survival without immunogenicity change. *Mouse models and clinical samples (melanoma and breast).*	(102)

Resistance to IFN-γ-dependent immunity is organized here not as a catalogue of lesions but along a single axis of escalating permanence. *Reversible gating* (A–C) lowers the amplitude of a structurally intact signal at the receptor (metabolism), transcriptional access (chromatin and RNA modification), and pre-existing oncogenic or environmental wiring upstream of JAK–STAT. *Hard-wired escape* (D) marks the point where selection fixes the phenotype into the genome, severing either signal reception (JAK1/2) or target display (B2M). *Clonal dynamics* (E) determine which of the many selectable routes is actually taken, because a lesion’s fitness is set by its ecological and clonal context. Selective value is set by clonal context, so the same lesion may indicate sensitization when clonal and resistance when subclonal; therapies acting on tumor-intrinsic IFN-γ signaling cannot reach the mutant fraction (Section 11.2).Induced phenotypic states (F) form a reversible, non-genetic axis in which cells change identity around the signal rather than silencing it; unlike blocks A–E, this block is not uniformly a resistance category, since two of its entries are associated with improved recognition or outcome. Throughout, the unifying claim is these mechanisms occupy one continuum from a reversibly restricted signal to a fixed one, and that reversibility maps onto therapeutic re-openability. Blocks A–E describe states enriched or maintained because they favor the tumor; block F describes states induced by the signal itself, whose consequence for tumor control must be established case by case rather than assumed.

The biosynthetic state converges on the same receptor node. In colorectal cancer, loss of bisecting N-glycosylation, via downregulated MGAT3, destabilizes the receptor chain IFNGRα through proteasomal degradation, producing intrinsic IFN-γ resistance reversible by restoring MGAT3 or all-trans retinoic acid (77). A broader glycolytic state may compound this: PFKFB3 was elevated in colorectal tumors and in PD-L1-blockade-resistant patients with shorter survival, and its suppression reduced tumorigenesis and improved immunotherapy, although these associations were largely correlative (78). Read together, metabolism behaves as a responsiveness threshold rather than a passive backdrop, set chiefly by IFN-γ-receptor abundance, which glutamine supply and N-glycosylation each control, and modulated by nutrient effects on effectors and glycolytic state. The implication is a metabolic, not genetic, layer of resistance: checkpoint-blockade responsiveness can vary even where the canonical apparatus is intact. This positions metabolism as one control point among several, alongside the genetic and epigenetic determinants of resistance and tumor selection considered next.

#### 5.1.2 Epigenetic gating of the IFN-γ/antigen-presentation axis

Reception is not an execution. Even when the IFN-γ receptor and kinases are intact, the transcriptional output depends on whether chromatin and RNA regulatory states permit IFN-γ-responsive and antigen-presentation genes to be read, making epigenetic and epitranscriptomic accessibility a gating layer. Resistance can therefore arise from restricted access to these programs rather than any signaling defect, and because such restriction is, in principle, reversible, reprogramming may reopen what evolution has closed. Chromatin sets a ceiling on antigen presentation. In melanoma, PRC2/EZH2 H3K27 hypermethylation suppresses tumor-specific MHC-II and its IFN-γ inducibility, potentially by reducing accessibility at CIITA, the MHC-II master regulator; PRC2 expression is inversely associated with anti-PD-1 response, and EZH2 inhibition restores MHC-II expression (79). Reopening these states has clinical traction: in ICB-refractory head and neck cancer, low-dose 5-azacytidine raised the IFN-γ signature and PD-L1 with more CD8^+^ and fewer Tregs, correlating with prolonged survival on rechallenge in a small trial (80). Pharmacological amplification also works: HSP90 inhibition upregulates interferon-response genes required for enhanced T-cell killing, potentiating checkpoint blockade (81).

The same logic extends from DNA and histones to RNA. Inhibiting the m6A methyltransferase METTL3 triggers an interferon response within tumor cells, whereas loss of the m6A reader YTHDF2 prevents MHC-I degradation. Together with strengthened T-cell IFN-γ and dampened exhaustion, this positions METTL3 as an epitranscriptomic checkpoint (82). A parallel mechanism operates on the immune side: in NAFLD-associated hepatocellular carcinoma, METTL3 restrains IFN-γ^+^ CD8^+^ T-cell infiltration via cholesterol metabolism, and targeting it synergizes with anti-PD-1 (83). Across chromatin, DNA methylation, chaperone-linked, and RNA-modification layers, one principle recurs: the amplitude of an IFN-γ response is set by transcriptional and epitranscriptomic accessibility, not reception alone; therefore, antigen presentation can fail with preserved upstream signaling. Because these states are pharmacologically reversible by EZH2, DNMT, HSP90, or METTL3 inhibition, restricted programs can be reopened and checkpoint-blockade sensitivity partially restored (**Figure 5**). Thus, epigenetic gating is reversible, distinct from the structural genetic disruptions of IFN-γ signaling.

**Figure 5:**
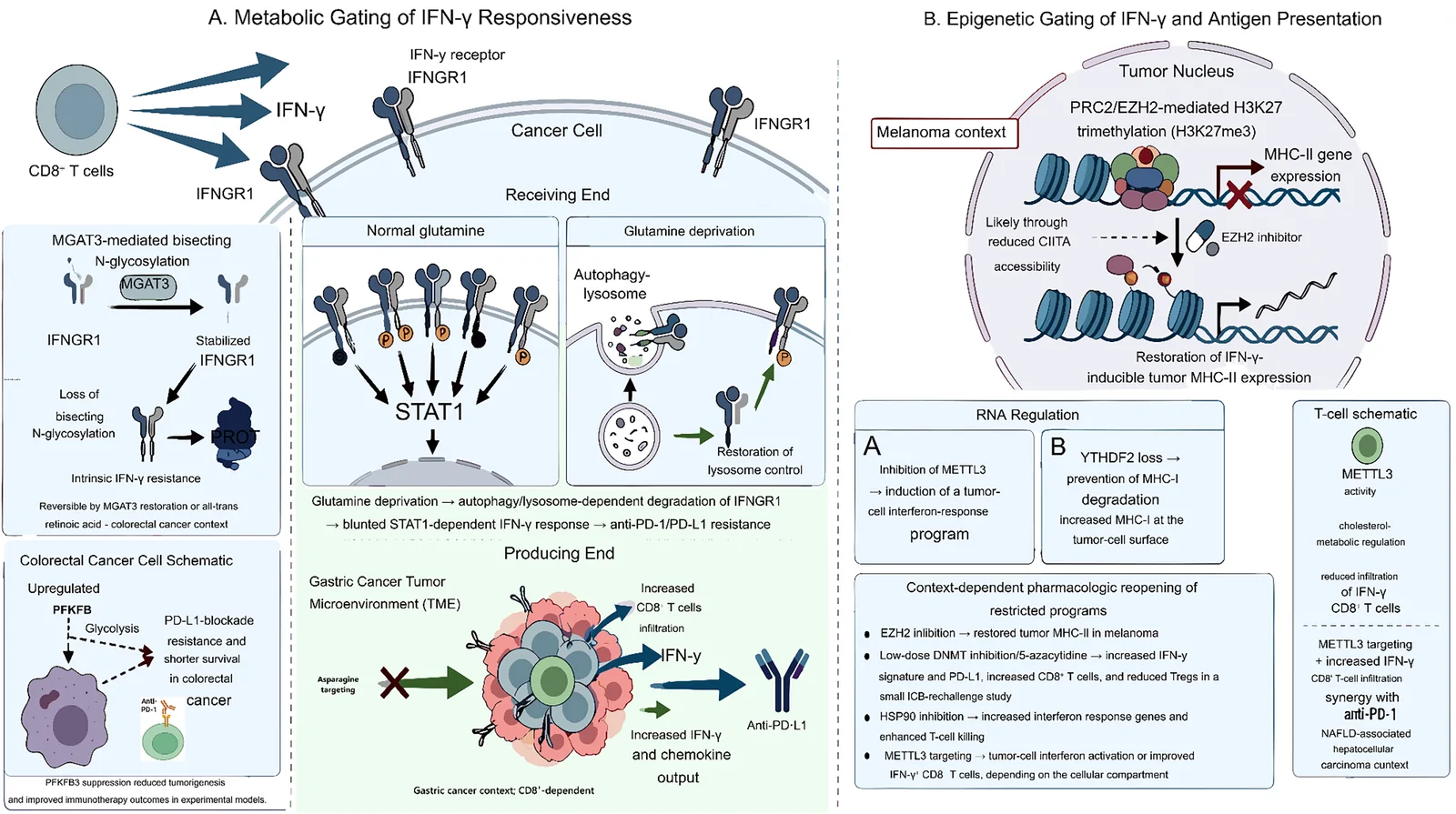
Metabolic and epigenetic gating of IFN-γ responsiveness and antigen presentation. **(A)** Metabolic regulation controls both the receiving and producing arms of IFN-γ signaling. Glutamine deprivation promotes autophagy/lysosome-dependent degradation of IFNGR1 and blunts STAT1-dependent signaling, whereas MGAT3-mediated bisecting N-glycosylation stabilizes IFNGR1 in colorectal cancer cells. A PFKFB3-associated glycolytic state is linked to PD-L1-blockade resistance, while asparagine targeting enhances CD8^+^ T-cell infiltration, IFN-γ and chemokine production, and anti-PD-L1 activity in gastric cancer. **(B)** Chromatin and RNA-regulatory mechanisms restrict IFN-γ responses and antigen presentation. PRC2/EZH2-mediated H3K27me3 suppresses IFN-γ-inducible tumor MHC-II expression, likely through reduced CIITA accessibility, whereas EZH2 inhibition restores this program. METTL3 inhibition induces a tumor-cell interferon-response program, YTHDF2 loss preserves surface MHC-I, and METTL3 targeting can increase IFN-γ^+^ CD8^+^ T-cell infiltration and enhance anti-PD-1 activity in NAFLD-associated hepatocellular carcinoma. These mechanisms are context dependent and represent potentially modifiable, non-genetic determinants of immunotherapy responsiveness.

#### 5.1.3 Oncogenic and environmental gating

Tumor cells do not act as neutral IFN-γ recipients. Their pre-existing oncogenic wiring and environmental exposures set a baseline competence that determines how far an IFN-γ signal travels before it is read or terminated. These inputs act upstream of canonical signaling, gating the probability of productive engagement rather than corrupting the pathway itself. Notably, several converge on the same JAK–STAT core through different routes. This convergence is significant. In melanoma, MYC represses JAK2 through an E-box in its promoter, lowering IFN-γ responsiveness, which is reversed by silencing MYC or MAX (84). In lung cancer, EGFR activation drives SHP2 to accelerate STAT1 dephosphorylation, prematurely terminating the IFN-γ response and curbing chemokine and antigen-presentation output; SHP2 inhibition restores sensitivity (85). Through a separate route, an IFN-γ-induced miR-1246–AGO2 complex stabilizes PTPN6 mRNA to dampen STAT1 phosphorylation and ISG and HLA output, again reversibly (86).

Extrinsic inputs can impose the same constraint. Tumor-infiltrating microplastics, isolated from colorectal tumors and blood, inhibited JAK–STAT signaling, downregulated IFN-γ, CXCL9/CXCL11, and the antigen-presentation genes B2m and H2-K1, reduced T-cell infiltration, and induced gut microbiota dysbiosis, worsening immunotherapy resistance in animal models (87). The fact that an environmental pollutant and several tumor-intrinsic regulators can restrict the same axis underscores the variability of these upstream inputs. Collectively, these mechanisms establish a baseline gate: a responsiveness ceiling or floor set before IFN-γ arrives, so that immunotherapy outcomes can be shaped by pre-existing tumor wiring and exposure independent of cytokine abundance. Because most operate by modulation rather than mutation, at JAK2, STAT1, or through the microenvironment, they are in principle reversible, distinguishing them from the irreversible, selected pathway disruptions taken up next, where immunoediting fixes resistance into the genome.

### 5.2 Tumor evolution under IFN-γ pressure: selection and induced adaptation

Immune pressure alters tumor populations through two mechanistically distinct routes, which are distinguished in this section. Selection enriches cells that already differ from their neighbors, whether through a genetic lesion (Section 11.1) or a pre-existing transcriptional state, with the outcome set by relative fitness in a particular clonal and ecological context (Section 11.2). Induction changes cells that did not previously differ, with IFN-γ itself driving transitions in differentiation status, antigen presentation, and survival behavior (Section 11.3). The two differ in whether the underlying change is heritable, whether it is reversible, and, importantly, whether it favors the tumor. A selected loss-of-pathway lesion is by definition advantageous under immune pressure, since that is why it was enriched. An induced state carries no such presumption, and IFN-γ-induced states have been reported with adverse, neutral, and favorable associations with patient outcome. We therefore treat induced plasticity as a consequence of IFN-γ exposure whose direction is context-dependent, rather than as an escape mechanism by default.

#### 5.2.1 Genetic loss-of-pathway escape

Sustained IFN-γ signaling does not merely cull susceptible tumor cells; over time, it reshapes the genetic composition of the population that survives. The clones that persist under prolonged immune attack are disproportionately those least able to sense, transduce, or execute IFN-γ-dependent programs, and recurrent loss-of-function lesions in this axis mark the point at which the constraint phase hardens into evolutionary escape. Genomic defects in IFN-γ pathway genes are enriched in tumors that fail anti-CTLA-4 therapy, and experimental IFNGR1 knockdown is sufficient to blunt rejection (7), positioning pathway integrity as a determinant rather than a passive correlate of response, although, as Section 11.2 develops, whether its loss favors or disfavors the tumor depends on the clonal context in which that loss occurs.

Disruption converges to the IFNGR–JAK–STAT cascade. Inactivating JAK1/2 mutations underlie both primary resistance, where they abolish reactive PD-L1 induction and the response to IFN-γ (88), and acquired resistance, where biallelic JAK1/JAK2 loss, together with insensitivity to the antiproliferative effects of the cytokine, emerges in relapsing lesions after initial regression (6). These two kinases are not interchangeable: JAK1 appears to be the principal conduit for IFN-γ-driven MHC, PD-L1, and cytostatic responses, whereas JAK2 is largely dispensable for most IFN-γ effects (89). This dependency may explain why JAK2 loss, frequently co-deleted with CDKN2A, heightens susceptibility to resistance (90) without necessarily extinguishing signaling. These lesions span a graded spectrum, from partial attenuation to complete signal reception severance.

The second convergent route disables the output rather than the input. Truncating B2M mutations and B2M loss of heterozygosity, enriched in non-responders across anti-CTLA-4 and anti-PD-1 cohorts (6, 91), collapse surface MHC-I and uncouple recognition from elimination. Whether a tumor ceases to hear the signal or to display its target, the phenotype converges on diminished vulnerability to IFN-γ-mediated control. Critically, the available evidence is most consistent with enrichment and clonal fixation of pre-existing or sporadically arising variants under persistent selection, rather than direct mutagenesis by IFN-γ itself; that such genetically defined resistance can be circumvented by rerouting immunity through NK and innate effectors (92) underscores its selective, not absolute, character. This type of selection can also be consolidated over time. In melanoma patient-derived models, acquired IFN-γ resistance was frequently initiated by JAK1/2 lesions detectable even before treatment and subsequently matured into HLA-class-I-negative lesions in which antigen-presentation genes were silenced and were no longer IFN-γ-inducible (93). The genetic lesion is the initiating event, and epigenetic silencing is its consolidation; therefore, this trajectory belongs to genetic escape rather than the reversible states considered in Section 11.3. Therefore, genetic escape represents the largely irreversible terminus of selection. However, not all durable resistance is hardwired, which directs attention to reversible, non-genetic states that allow tumors to weather the same pressure without fixing a single mutation.

#### 5.2.2 The selectable landscape and clonal dynamics of escape

If genetic escape (Section 11.1) identifies the lesions that survive immune pressure, it does not, by itself, explain which of the many possible escape routes a tumor will take. Resistance is better understood as a property of a population rather than a cell: IFN-γ-mediated selection acts upon a heterogeneous landscape of potentially selectable states, and which of them become enriched depends on their fitness within a particular clonal and ecological context. Functional genomic screens have begun to chart the dimensions of this landscape. *In vivo* CRISPR screens recover canonical IFN-γ-pathway and antigen-presentation determinants while also nominating regulators such as PTPN2, whose loss enhances rather than impairs IFN-γ-mediated effects (95), and they reveal that the consequences of tumor IFN-γ sensing are themselves double-edged, since loss of tumor IFN-γ signaling can sensitize some models through NK- and NKG2A-dependent mechanisms (96). Other screens identify modifiers such as CHD1 and MAP3K7 that tune, rather than abolish, the IFN-γ response (97). Collectively, these maps define a space of potentially selectable routes; crucially, they enumerate what could be selected, not what necessarily is, since actual evolutionary outcomes depend on biological context and relative fitness.

That contingency is sharpest where clonal architecture intervenes, and the observation warrants more than passing mention because it inverts the expected direction of selection. In co-culture, loss of *Ifngr2* or *Jak1* protected B16.SIY melanoma cells from T-cell-mediated killing, a result that would predict the resistance literature. Implanted as pure populations, however, the same mutant tumors were controlled better than wild-type tumors because they could no longer upregulate PD-L1 and thus forfeited their principal defense against CD8^+^ T cells. Outgrowth of the mutant cells occurred only when they were admixed with wild-type cells and depended on PD-L1 expression by those wild-type neighbors (5). Therefore, the lesion is not intrinsically advantageous; its selective value is conferred by the population surrounding it.

This helps resolve a discrepancy that runs through the wider literature, and that Sections 11.1 and 11.2 would otherwise leave unreconciled. A meta-analysis of 33 functional genomic screens found alterations in IFN-γ signaling genes enriched under immune pressure in 17 of 19 *in vitro* co-culture screens but significantly depleted in 9 of 14 *in vivo* screens, and across 29 clinicogenomic studies comprising 2,154 patients with solid tumors from seven tissues, tumor-intrinsic mutations in *JAK1*, *JAK2*, *IFNGR1*, *IFNGR2*, and *STAT1* correlated with a greater rather than lesser likelihood of response to checkpoint inhibition (103). The authors noted that their conclusions diverge from earlier reports and call for larger cohorts, and the documented cases of acquired resistance emerging in relapsing lesions (6, 88) are not in doubt. Clonal cooperation supplies a plausible reconciliation: whether an IFN-γ-pathway lesion helps or harms the tumor depends on whether PD-L1-competent cells persist alongside it, so the same mutation may indicate sensitization when clonally dominant and resistance when it arises as a minority subclone within a signaling-competent tumor. This interpretation is consistent with, though not directly tested by, the clinicogenomic analysis, in which pre-treatment variants detected by bulk sequencing would tend to be clonal.

Parallel observations in patients associate explosive tumor outgrowth with concurrent neoantigen erosion and diminished IFN-γ signaling (98), suggesting that simultaneous collapse of recognition and responsiveness may accelerate escape. Five clinical implications follow. First, an IFN-γ-pathway lesion is not a stand-alone resistance biomarker: its effect depends on clonal context and on the pathway competence of neighboring cells, features that variant allele frequency alone cannot resolve (5). Second, tumor-intrinsic strategies that enhance IFN-γ signaling (104–106) have a corresponding coverage limit: cells lacking an intact pathway would not be expected to respond through that mechanism, so their efficacy in a heterogeneous tumor should depend partly on the fraction of pathway-competent cells. Third, because wild-type neighbors can shield IFN-γ-insensitive cells through PD-L1 (5), increasing IFN-γ signaling across a heterogeneous tumor could, in principle, reinforce this protection. This remains an untested inference. Fourth, strategies that do not require intact tumor IFN-γ signaling may therefore be preferable for addressing resistant subclones. TLR9 agonism combined with PD-1 blockade overcame JAK1/2-dependent resistance through NK- and CD8 T-cell activity (92), while restoring antigen presentation independently of IFN signaling provided another route around JAK1 loss (107). JAK inhibition also selectively suppressed IFN-γ-pathway-deficient melanoma through T-cell- and host TNF-dependent mechanisms (108). Thus, in heterogeneous tumors, combining pathway-dependent and pathway-independent mechanisms may provide broader coverage than either alone. One further possibility warrants flagging as untested: because PD-L1 is also exported on exosomes and IFN-γ increases the amount carried on them (70), the shielding effect might extend beyond the direct cell contact demonstrated by Williams and colleagues (5), which would widen the radius over which a pathway-competent clone can protect an incompetent one. All in all, these findings recast immune escape as an ecological process: selection filters a diverse landscape, and clonal interactions can render its trajectory non-linear and occasionally abrupt, behavior the IFN-γ Response Cycle must accommodate at the system level.

#### 5.2.3 Induced phenotypic states: dedifferentiation, antigen remodeling, and the stem-like state

Sections 11.1 and 11.2 concern variants that differ from their progenitors and are enriched because this difference confers a fitness advantage. A second class of state change requires no such pre-existing difference; IFN-γ can itself induce transitions in lineage identity, differentiation status, and antigen visibility in genetically competent cells. These are adaptations rather than selected variants, and they are not uniformly adverse.

The clearest illustration is dedifferentiation, which runs counter to the expected direction. Continuous IFN-γ exposure drove differentiated melanoma lines toward a neural-crest-like state through global chromatin remodeling; in the same study, biopsies from patients who responded to anti-PD-1 showed decreased melanocytic and increased neural-crest markers, and the IFN-γ-induced dedifferentiation signature corresponded with improved outcomes (94). The authors framed this as challenging the view that neural-crest dedifferentiation is entirely an adverse phenotype, and we adopt that reading: IFN-γ-induced dedifferentiation is better understood as a marker of an engaged immune response whose consequence for tumor control is context-dependent than as an escape phenotype. Antigen remodeling behaves similarly. Prolonged IFN-γ acting through IDO1-mediated tryptophan depletion provoked ribosomal frameshifting and the surface presentation of aberrant trans-frame peptides; priming of naive T cells with these peptides generated peptide-specific T cells, leading the authors to conclude that this diversification of the peptidome contributes to immune recognition of melanoma cells (100). Neither observation describes escape, and we cite them here as evidence that IFN-γ-induced state change is directionally variable.

Other induced states carry demonstrated escape consequences. In breast cancer models, IFN-γ directly converts non-stem to stem-like cells through BCAT1, conferring resistance to cytotoxic killing and metastatic competence, and BCAT1 blockade improves the efficacy of vaccination and checkpoint blockade (16). On a much shorter timescale, IFN-γ-activated T cells induced transient cell-in-cell structures via STAT3 and EGR-1 that shielded inner cells from killing and reseeded tumors once T cells withdrew, conferring survival without any change in immunogenicity (102). These are genuine instances of the productive signal generating its own constraint. Both are also reversible, persisting only while the inducing pressure is applied, which distinguishes them mechanistically from the fixed lesions of Section 11.1.

A third group is observed at or before progression but has not been resolved as selection or induction. A dedifferentiated MITF-low/AXL-high phenotype with transcriptional MHC-I downregulation marked melanomas progressing on PD-1 blockade, but was attributed to TGF-β rather than IFN-γ (99). A TCF4-centered regulatory network enforcing a mesenchymal-like, antigen-presentation-low program was enriched in early non-responder biopsies, and its presence at that stage argues for a pre-existing state that is selected rather than one induced by therapy; TCF4 inhibition through BET blockade restored immunogenicity (101). In neither case were tumor cells tracked longitudinally at single-cell resolution during treatment, so whether the responsible population expanded or converted remains unresolved.

Taken together, Sections 11.1 to 11.3 describe two processes that are frequently reported under the single heading of escape but should not be equated. Selection acts on heritable differences and, where those differences are genetic, produces the largely irreversible loss of pathway integrity described in Section 11.1. Induction acts on genetically competent cells and produces reversible states whose consequences range from enhanced recognition (94, 100) to unresolved (99, 101) and durable survival under attack (16, 102). Conflating them overstates the case for plasticity as an escape route while understating its potential value as a marker of immune engagement. For the Cycle, the operational consequence is that only the first process is expected to be irreversible: induced states remain in principle therapeutically re-openable, which is the premise of the interventions considered in Section V.

PART V. BREAKING THE CYCLE (Cut-Points): Decoupling Killing from Selection.

12. Restoring and rewiring the signal: re-sensitization and selective uncoupling.

If Sections 8–11 trace how the IFN-γ Response Cycle fails, the converse question is whether this failure is reversible. The studies gathered here suggest that resistance is frequently a state to be reopened rather than an endpoint, and, importantly, that the most promising interventions do not simply add more IFN-γ but restore, bypass, or selectively reshape how its programs are executed. The unifying therapeutic aim is thus to short-circuit the counter-regulatory feedback that IFN-γ itself sets in motion, preserving its cytotoxic and antigen-presenting output while withholding the checkpoint-induction and selective programs that follow, rather than to intensify the signal indiscriminately. **Table 2** organizes the strategies considered across this Part by their cut-point in the IFN-γ Response Cycle rather than by drug class, together with the platform in which each was tested and the level of evidence available.

**Table 2:** Breaking the cycle, therapeutic strategies mapped to their cut-point in the IFN-γ Response Cycle.

Strategy class	Cut-point	Mechanistic rationale	Evidence	Refs.
A. Re-sensitization and selective uncoupling — cut-point: reverse the Turn/reopen Adaptation (2→3, 3→4)				
Phosphatase degradation	2→3 *re-sensitize*	Targeted degradation of TC-PTP (PTPN2) alone or in combination with PTP1B releases intracellular brakes on JAK–STAT1, enhancing antigen presentation and effector function.	Preclinical *(in vivo; one in vitro only)*	(104, 105)
Selective arm uncoupling	2→3 *uncouple*	ULK1 binds to IRF1 and shapes PD-L1 promoter occupancy; its inhibition dampens IFN-γ-induced immunosuppressive genes while sparing immunostimulatory ones.	Preclinical *(in vivo)*	(106)
Bypassing or exploiting a broken pathway	3→4 *bypass/exploit*	Restoring antigen visibility independently of IFN-γ signaling (MDM2 inhibition → p53 → IL-15/MHC-II; NLRC5 or dsRNA sensing → NF-κB → MHC-I in JAK-loss tumors) or converting the resistance lesion itself into a vulnerability (constitutive JAK1/2 activity in IFNGR1-deficient tumors).	Preclinical *(in vivo)*	(107–109)
B. Rational checkpoint combinations — cut-point: govern the Turn (2→3); distribute activation across productive and self-limiting arms				
Non-redundant checkpoint arms	2→3 *govern*	Anti-CTLA-4 and anti-PD-1 induce largely non-overlapping transcriptional and functional signatures (memory-proliferative versus cytolytic/NK), engaging complementary arms of immunity.	Clinical (correlative) *45 patients; blood and tumor profiling*	(110)
IFN-γ layered onto PD-1 blockade	2→3 *govern*	Tolerable in combination; monocyte PD-L1 increased with IFN-γ dose and duration — a clinical observation of the feedback the cycle predicts.	Clinical (trial) *Phase 1, solid tumors, n = 26*	(17)
Innate co-activation and dose optimization	2→3 *govern/optimize*	Coupling STING activation to checkpoint release: mathematical modeling that formalizes the productive-versus-feedback trade-off and defines a threshold dose.	Preclinical (*in vitro*); in silico	(111, 112)
C. STING and innate-sensing ignition — cut-point: ignite from upstream (→ Node 1→2); innate-to-adaptive, compartment-dependent				
Innate ignition of the adaptive loop	→1→2 *ignite*	Activated T-cell cytokines sensitize tumor cells to STING activation, amplifying type-I IFN and reinforcing the cytotoxic T-cell state. Oncolytic STING signaling raises tumor MHC-I and, predictably, PD-L1.	Preclinical *(in vivo)*	(113, 114)
Compartment-selectivity is required	→1→2 *selective*	The benefit derived from cancer cell-intrinsic STING, whereas T cell-intrinsic STING was associated with exhaustion; a STING–TOX loop repressed HO-1 and triggered ferroptosis in tumor-infiltrating CD8^+^ T cells.	Preclinical *(in vivo)*	(115, 116)
D. Spatiotemporally controlled IFN-γ delivery — cut-point: set space, time, and intensity of Execution (1→2) while constraining the feedback Turn				
Materials setting dose and duration	1→2 *set dose/kinetics*	Mineral-coated mRNA capture-and-recycle, PEGylated liposomes, TLR-activating chitosan/γ-PGA nanoparticles, and antigen–plasmid co-delivery liposomes extend or confine the active window.	Preclinical *(in vivo)*	(117–120)
Cellular and microbial carriers	1→2 *localize*	Tumor tropism exploited for local IFN-γ production: macrophage mRNA vehicles, DC-programming nanoparticles, engineered BMSCs co-expressing soluble PD-1, ADSCs ± TRAIL, attenuated Salmonella, and recombinant BCG.	Preclinical *(in vivo)*	(121–126)
Delivery designed to constrain feedback	1→2 *limit feedback*	Co-delivery, in which triptolide reverses IFN-γ-inducible PD-L1 within a hydrogel, is the only delivery strategy that addresses the feedback arm rather than localization alone.	Preclinical *(in vivo)*	(127)
E. Cell-based therapies leveraging IFN-γ output — cut-point: sustain Execution (1→2) via a renewable, sensing effector source				
Renewable effectors, IFN-γ as input or readout	1→2 *sustain*	Low-dose IFN-γ priming to broaden TIL reactivity; off-the-shelf CAR-NK manufacture using IFN-γ as an effector-function readout; and a cytokine-armed oncolytic virus reinforcing CAR-NK activity.	Clinical (correlative) *for TIL priming*; otherwise, preclinical	(128–130)
Design rationale for heterogeneity-spanning executors	1→2 *sustain*	Conceptual argument for renewable sensing effector sources capable of spanning intratumoral heterogeneity	Conceptual *(review)*	(131)
F. Vaccines, dendritic-cell platforms, and adjuvants — cut-point: raise the probability of productive initiation (→ Node 1 → 1→2)				
Improving antigenic stimulus and DC licensing	→1 *prime*	Death modality shapes priming (ferroptotic lysates); maturation signals raise output (LPS-supplemented protocols); and engineered antigen carriers (thermostable nanoarchaeosomes).	Preclinical *(in vivo; one in vitro only)*	(132–134)
Relieving brakes on priming	→1 *de-repress*	AhR inhibition in dendritic cells promotes IL-12 and, in turn, NK cell IFN-γ; PI3K inhibition combined with a TLR agonist suppresses IL-10 and TGF-β, yielding polyfunctional T cells.	Preclinical *(in vivo)*	(135, 136)
Engineered delivery for breadth and durability	→1 *prime*	Neoantigen nanocomplexes, α-GalCer-modified lipid nanoparticles engaging CD8^+^ and iNKT cells in parallel, retargetable IFN-γ-mRNA extracellular vesicles, hypoxia-relieving nanovaccines, DC-aptamer-decorated exosomes, and compact gene activation toolkits.	Preclinical *(in vivo; one model is non-oncologic)*	(137–142)
G. Ferroptosis–immunotherapy synergy platforms — cut-point: weld Execution back to Initiation (1→2 ↔ init); bidirectional IFN-γ–ferroptosis loop				
Platforms coupling ferroptotic death to IFN-γ output	1→2 ↔ init *reinforce*	ROS- and arachidonic acid-supplying nanosystems, MOF@COF nanovaccines, adjuvant-supplying stimuli-responsive liposomes, TIGIT-blocking hemin, NK-exosome/RSL3 hybrid vesicles, and blood–brain-barrier-crossing bacterial outer-membrane vesicles.	Preclinical *(in vivo)*	(143–148)
The closed-loop design concept and its limits	1→2 ↔ init *concept*	The explicit design goal is to achieve mutual reinforcement between ferroptosis and immunotherapy. Nonselective ferroptosis can be immunosuppressive, which is why targeting and adjuvant pairing are considered central rather than incidental.	Conceptual *(review)*	(149)
H. Microenvironment reprogramming and physical or chemotherapeutic combinations — *cut-point: condition the stage toward an IFN-γ-permissive state (upstream of 1→2)*				
Physical modalities inducing immunogenic death	upstream *condition*	Sonodynamic anti-PD-L1 nanobubbles, ultrasound-switched engineered bacteria, photodynamic therapy with abscopal control, and a photothermal bispecific nanomodulator that neutralizes the feedback provoked.	Preclinical *(in vivo)*	(150–153)
Metabolic, stromal, and vascular conditioning	upstream *condition*	GLUT1 blockade limits lactate excretion, CRISPR-mediated PKM2 disruption curtails the Warburg effect, decorin-mediated stromal conditioning (β2M/CD8-dependent), and VEGFR2 nanoliposomal vaccination with paclitaxel.	Preclinical *(in vivo)*	(154–157)
Supplying and preserving targets alongside chemotherapy or blockade	upstream *condition*	IFN-γ with chemotherapy raises MHC-I in cold tumors, ALP-responsive co-delivery of IFN-γ and doxorubicin, PD-L1 self-blockade outer-membrane vesicles, immunogenic-death-amplifying payloads in KRAS-mutated tumors, and oncolytic reovirus combined with vaccine and checkpoint blockade.	Preclinical *(in vivo)*	(158–162)

Strategies are organized according to where they intervene in the IFN-γ Response Cycle, rather than by conventional drug class. Re-sensitization and selective uncoupling (A) reopen or bypass impaired signaling and separate beneficial from resistance-associated outputs; checkpoint combinations (B) modulate the productive-to-self-limiting Turn; and STING and other innate-sensing strategies (C) initiate the cycle upstream. Controlled delivery and cell-based platforms (D–E) regulate the spatial, temporal, or cellular context of IFN-γ execution, whereas priming strategies, ferroptosis–immunotherapy synergy platforms, and microenvironmental reprogramming (F–H) promote productive initiation, reinforce execution–initiation coupling, and establish an IFN-γ-permissive state. Overall, the framework emphasizes steering where and how IFN-γ signaling operates, rather than simply maximizing its abundance. Most evidence remains preclinical, and clinical, in silico, and conceptual studies are indicated accordingly.

The first principle is the release of intracellular brakes. Several tumor-intrinsic regulators restrain IFN-γ signal transmission, and removing them can re-establish responsiveness. Selective or dual degradation of the phosphatases TC-PTP and PTP1B elevates STAT1/JAK phosphorylation, intensifies IFN-γ signaling, and increases MHC-I expression, while also activating T-cell and CAR-T effector function in the models tested (104, 105). A complementary, more selective logic targets the downstream apparatus rather than the cascade itself: ULK1 appears to bias IFN-γ output toward its immunosuppressive arm, binding IRF1, and influencing PD-L1 promoter occupancy, so that ULK1 inhibition preferentially dampens IFN-γ-induced immunosuppressive genes with minimal effect on immunostimulatory ones (106). Direct experimental evidence that these IFN-γ outputs can be separated comes from structure-guided engineering of partial, biased IFN-γ agonists: these variants preserved MHC class I upregulation while disproportionately reducing PD-L1 induction, with MHC-I bias observed across seven human cancer cell lines, although to varying degrees among cell types (163). These findings provide proof-of-principle that selected immunostimulatory and immunosuppressive outputs of IFN-γ signaling can be partially decoupled at the ligand–receptor level. This distinction is important: amplification raises all outputs, whereas uncoupling separates beneficial from resistance-associated programs.

This uncoupling logic extends to antigen presentation, and the output is most often lost. MDM2 inhibition, through p53 induction, can increase IL15 and MHC-II levels and is associated with antitumor immunity and memory in melanoma models, reopening immune visibility largely independent of the IFN-γ cascade (109). When the cascade is structurally broken, presentation can be reconstituted by bypassing. In JAK1-deficient melanoma, restoring MHC-I via NLRC5 overexpression or dsRNA-sensor activation by BO-112, acting through NF-κB independent of IFN signaling, restores the efficacy of adoptive cell therapy (107). Restored MHC-I is sufficient for ACT in these models, although it does not equate to a clinical response. Finally, deficiencies can become liabilities. IFNGR1-deficient melanomas acquire constitutively active JAK1/2, rendering them selectively vulnerable to JAK inhibition in a T cell-and host TNF (108). Together, these findings suggest a unifying therapeutic grammar: resistance may be re-sensitized, bypassed, redirected, or even exploited, rarely by demanding more IFN-γ, more often by changing how, where, and which of its programs are run. These strategies share a structural constraint that follows from the clonal cooperation data in Section 11.2: re-sensitization, uncoupling, and amplification each act on cells that retain a functional IFN-γ pathway, and none can reach a subclone in which that pathway is genetically lost. Therefore, their expected benefit in a heterogeneous lesion is bounded by the pathway-competent fraction, which argues for pairing them with effectors that do not depend on tumor IFN-γ signaling (92, 107).

### 5.3 Checkpoint combinations and innate-sensing ignition

#### 5.3.1 Rational combinations with checkpoint blockade

Combination therapy is often framed as additive immune activation; however, the IFN-γ Response Cycle reframes it as an attempt to govern the timing, magnitude, and downstream consequences of that activation. The relevant question is less about which agents are paired than about how their effects are distributed across the cycle’s productive and self-limiting phases. The fact that checkpoint agents are not mechanistically interchangeable is itself instructive: anti-CTLA-4 and anti-PD-1 induce largely non-overlapping transcriptional and functional signatures *in vivo*, proliferative programs in memory T-cell subsets versus cytolytic and NK-associated programs; therefore, their combination engages complementary, rather than redundant, arms of immunity (110). Layering IFN-γ directly onto checkpoint blockade is feasible: a phase 1 study of IFN-γ with nivolumab established tolerability and, notably, observed that monocyte PD-L1 rose with IFN-γ dose and duration (17), a clinical readout of the feedback the cycle predicts, situating the productive and restraining effects of IFN-γ on a single dose- and time-dependent axis.

Coordinating adaptive release with innate sensing offers another axis of control. In gastric cancer, IFN-γ combined with a STING agonist and PD-1 blockade was associated with elevated cGAS-STING expression, IFN-β production, and increased apoptosis, suggesting the integration of innate activation with checkpoint release (111), although this evidence rests largely on expression and co-culture data rather than clinical outcomes. Where empirical pairing reaches its limits, systems-level modeling can formalize the trade-off. A mathematical model incorporating both pro- and anti-inflammatory IFN-γ functions reproduced the observation that tumor reduction under anti-PD-1 treatment improves as IFN-γ production efficiency decreases and defined a threshold dosage relating anti-PD-1 to IFN-γ output (112). Such predictions are theoretical rather than clinically validated, yet they articulate the central design problem, identifying windows in which productive IFN-γ signaling is maximized before feedback-associated restraint dominates, and motivate strategies that more directly ignite innate sensing, considered next.

#### 5.3.2 STING and innate-sensing combinations feeding the IFN-γ axis

If checkpoint combinations modulate IFN-γ biology from within the adaptive compartment, innate sensing offers a way to ignite it from upstream. STING activation can feed the IFN-γ axis, yet the evidence assembled here cautions that its consequences depend on where and in which cells signaling occurs. A recurring and productive motif is a reciprocal innate–adaptive loop. In T-cell-engaging therapy for AML, a STING agonist enhances bispecific-antibody-mediated cytotoxicity because activated T cells, through IFN-γ and TNF, render tumor cells more susceptible to STING activation, amplifying type I IFN and IFN-stimulated genes that feed back to reinforce the cytotoxic T-cell state (113). Oncolytic immunotherapy engages a comparable circuit: innate STING signaling in immune cells transmits an interferon response to tumor cells, which respond through JAK-STAT by upregulating MHC-I and, predictably, PD-L1, yielding a more inflamed phenotype and a rationale for anti-PD-1 combination (114). In both, IFN-γ operates as the connective signal linking innate ignition to adaptive execution.

However, the same pathway can be maladaptive when it fires in the wrong compartment. In CAR-T therapy against pancreatic models, the efficacy of a STING agonist requires genetic ablation of STING within the CAR-T cells themselves, since cancer-cell-intrinsic, not T-cell-intrinsic, STING drove benefit. In contrast, T-cell-intrinsic signaling is associated with exhaustion (115). Relatedly, a STING–TOX regulatory loop has been reported to repress HO-1 and trigger ferroptosis in tumor-infiltrating CD8^+^ T cells, such that STING- or TOX-deficient T cells display improved persistence and IFN-γ output (116). Identical innate sensing thus appears to support productive immunity in tumor or myeloid cells, while potentially impairing the very T cells it is meant to empower. Together, these observations suggest that effective innate-sensing strategies may hinge on selective activation, engaging beneficial tumor-extrinsic circuits while sparing T-cell-intrinsic ones, rather than on global STING enhancement. This principle frames the concluding synthesis of how the IFN-γ Response Cycle might be therapeutically steered.

### 5.4 Engineering the signal: controlled delivery and cell therapy

#### 5.4.1 Spatially and temporally controlled IFN-γ delivery (gene/protein/cell carriers)

The biology of IFN-γ is inseparable from its coordinates: the amount of signal reaching which cells, where, and for how long it acts. Delivery engineering can therefore be read not as a way to supply more cytokine but as an attempt to set the space, time, and intensity parameters of the IFN-γ Response Cycle directly, maximizing productive activation locally while constraining the systemic exposure that has limited free recombinant IFN-γ. Synthetic carriers pursue this along several axes. Persistence and potency are addressed by formulation: a nanostructured mineral coating that captures and recycles mRNA-expressed IFN-γ reportedly extends its active window and raises bioactivity relative to recombinant protein, lowering the dose required for macrophage reprogramming (117), whereas PEGylated liposomes prolong circulation and tumor accumulation compared with non-PEGylated counterparts (118). Spatial confinement and microenvironmental remodeling are achieved through TLR-activating chitosan/γ-PGA nanoparticles co-administered with IFN-γ (119) and through transiently expressed, locally restricted mRNA (121). A complementary approach is co-delivery, pairing IFN-γ with an antigen to focus CTL priming (120), or with agents that blunt its own feedback, as when triptolide reverses IFN-γ-inducible PD-L1 within a hydrogel system (127). Notably, such engineering is not uniformly additive; in one antigen–cytokine platform, physical complexation lowered transfection and merely matched, rather than exceeded, simpler co-injection (120).

Living carriers shift the strategy from delivering cytokines to generating them at the target. Endogenous macrophages can be co-opted as tumor-homing vehicles for IFN-γ mRNA, yielding transient intratumoral expression and M1 repolarization (121), and dendritic cells can be programmed ex vivo to produce IFN-γ through an IL-12/IL-18 autocrine circuit (122). Engineered mesenchymal and stromal stem cells exploit tumor tropism to localize IFN-γ, alone, with soluble PD-1, or with TRAIL, and were associated with reduced regulatory T cells and prolonged survival in lung and melanoma models (123, 124). Microbial platforms extend the same principle: IFN-γ sustained the persistence and antitumor activity of attenuated Salmonella while shifting the myeloid balance toward M1 macrophages (125), and recombinant BCG secreting IFN-γ upregulated MHC-I and improved survival in experimental bladder cancer (126). Across these otherwise disparate platforms, a shared intent emerges: to widen the therapeutic index by reshaping the geography and kinetics of IFN-γ exposure rather than its total amount. The evidence remains preclinical and platform-specific, and recurrent IFN-γ-driven PD-L1 induction is a reminder that localized delivery does not abolish the cycle’s feedback. These limitations point toward carriers that not only deposit IFN-γ but actively sense and respond to it, the engineered cellular therapies considered next.

#### 5.4.2 Cell-based therapies leveraging IFN-γ output

Where the carriers of Section 14.1 deposit a finite quantity of IFN-γ, cell-based therapies introduce something different: a living source that can sense its surroundings and produce IFN-γ continuously as an effector output. In these platforms, IFN-γ is read less as a biomarker than as the functional currency linking transferred cells to the broader remodeling of the tumor microenvironment. The cytokine occupies both ends of this relationship. As an input, low-dose IFN-γ pretreatment of autologous melanoma can sensitize tumor targets and broaden the reactivity of tumor-infiltrating-lymphocyte products, upregulating MHC and restoring immunogenicity, with high *in vitro* reactivity of the infusion product associated with clinical response (128). As an output, IFN-γ serves as a core readout of engineered effector function: off-the-shelf CAR-NK cells generated from leukoreduction-filter-derived NK cells produced IFN-γ and granzyme B upon target engagement at levels comparable to peripheral-blood counterparts, supporting their feasibility as a scalable source (129). NK- and T-cell platforms thus converge on IFN-γ as a shared signature of productive cytotoxic activation.

Combinatorial designs aim to amplify and sustain the output. Pairing anti-ROR1 CAR-NK cells with an IL-21-secreting oncolytic virus increased their IFN-γ, granzyme B, and perforin secretion and extended survival in neuroblastoma xenografts, illustrating how a cytokine-armed virus can reinforce cellular effectors (130). Such strategies are particularly motivated by intratumoral heterogeneity and the progressive disabling of T-cell and myeloid functions, as characterized in glioblastoma, which erode single-target approaches and favor heterogeneity-spanning designs (164). Interpreted through the IFN-γ Response Cycle, these therapies act as engineered, renewable executors capable of maintaining immune pressure and adapting to an evolving tumor; however, whether sustained IFN-γ output translates into durable clinical control remains to be established. This reframes IFN-γ not as a fixed agent but as a dynamically regulated system, the synthesis of which we now discuss.

### 5.5 Priming and amplifying the cycle from the productive end

#### 5.5.1 Vaccines, dendritic-cell platforms, and adjuvants

If the preceding strategies act on execution and constraint, vaccines and dendritic cell (DC) platforms intervene at the productive entry point of the IFN-γ Response Cycle: the priming step that determines whether IFN-γ-producing lymphocytes are generated. Across otherwise heterogeneous technologies, IFN-γ recurs as the common readout of successful priming, and two complementary approaches dominate: improving antigen presentation and relieving the suppression that limits it. The first concerns the quality of the antigenic stimulus and the licensing of DC that present it to T cells. The form of tumor-cell death shapes this input: DC vaccines loaded with ferroptotic glioma lysates elicited IFN-γ and durable CD8^+^ responses in a manner dependent on calreticulin and ATP rather than HMGB1–TLR4; however, supplementing those DAMPs into non-immunogenic lysates did not fully reconstitute efficacy, indicating that immunogenicity is not reducible to single danger signals (132). Maturation signals are important: adding LPS to the DC maturation protocol raised intratumoral IFN-γ and T-helper infiltration while lowering regulatory T cells in a breast cancer model (133), and engineered antigen carriers such as thermostable nanoarchaeosomes drove APC maturation, T-cell proliferation, and IFN-γ *in vitro* (134).

The second removes brakes on priming. Targeting aryl-hydrocarbon-receptor signaling within DCs promoted IL-12 and, in turn, NK cell IFN-γ and cytotoxicity (135), whereas PI3K inhibition combined with a TLR agonist suppressed IL-10 and TGF-β to yield polyfunctional T cells co-producing IFN-γ and IL-17, with protection abolished in the absence of either cytokine (136). A protein adjuvant has also been proposed as a precision immunotherapy to prevent recurrence after colorectal resection (165). Engineered delivery extends these principles to their breadth and durability. Neoantigen nanocomplexes co-delivered with chemotherapy increased CD8+IFN-γ+ cell infiltration in colorectal cancer models (137). α-GalCer-modified lipid nanoparticles engage CD8^+^ T cells and iNKT cells in parallel and sustain IFN-γ in draining lymph nodes (138). Retargetable IFN-γ-mRNA-loaded extracellular have demonstrated antitumor activity, even against intrinsically resistant glioblastoma (139). Strategies aimed explicitly at heterogeneity, oxygen-carrying nanovaccines that relieve hypoxia and reduce stemness (140), aptamer-directed DC-targeted exosomes (demonstrated as an immunomodulatory, IFN-γ-skewing platform in an allergic model) (141), and compact gene-activation toolkits inducing CXCL9, IL-15, and IFN-γ (142) seek to widen the antigenic and effector coverage (**Figure 6**).

**Figure 6:**
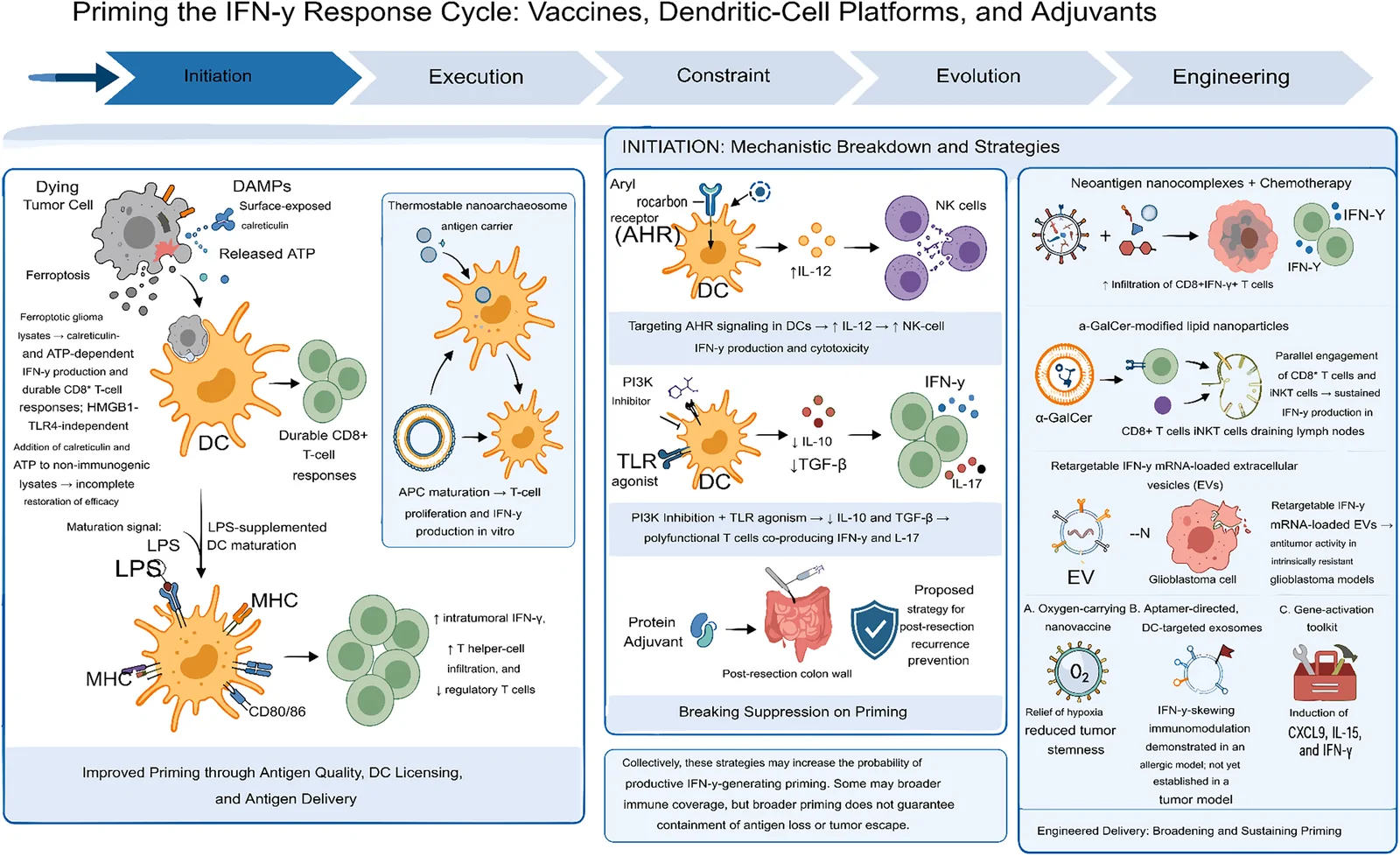
Priming the IFN-γ response cycle through vaccines, dendritic-cell platforms, and adjuvants. Productive initiation is strengthened by improving antigen quality, dendritic-cell licensing, and antigen delivery, including ferroptotic glioma lysates, LPS-supplemented DC maturation, and nanoarchaeosome-based antigen carriers. Suppressive barriers to priming can be relieved through AHR targeting, combined PI3K inhibition and TLR agonism, and protein-adjuvant strategies, thereby promoting IL-12-dependent NK-cell activity or polyfunctional T-cell responses. Engineered delivery platforms, including neoantigen nanocomplexes, α-GalCer-modified lipid nanoparticles, IFN-γ mRNA-loaded extracellular vesicles, oxygen-carrying nanovaccines, DC-targeted exosomes, and gene-activation toolkits—extend priming breadth and durability. These approaches are largely preclinical and platform-specific; although they may increase the probability of productive IFN-γ-generating immunity, broader priming does not necessarily prevent antigen loss or tumor escape.

Throughout the cycle, these approaches share a logic: raising the probability that a productive, IFN-γ-generating response is initiated, ideally with sufficient breadth to limit antigen loss and the escape-associated induced states cataloged earlier. The evidence is largely preclinical and platform-specific, and broader priming does not guarantee effective containment. By strengthening initiation, they complete a continuum- initiation, execution, constraint, evolution, and engineering- that the concluding synthesis now draws together into a single IFN-γ Response Cycle.

#### 5.5.2 Ferroptosis–immunotherapy synergy platforms

The IFN-γ–ferroptosis execution axis introduced earlier has found its most literal engineering in platforms built to reinforce tumor killing and immune activation. Their shared premise is mechanistic: CD8^+^ T-cell-derived IFN-γ suppresses the cystine–glutamate antiporter system x_c^-^ by downregulating SLC3A2 and SLC7A11, thereby restricting cystine uptake and promoting lipid peroxidation and ferroptosis in tumor cells (31). This provides a mechanistic basis for coupling immune activity to increased tumor ferroptosis. This bidirectional coupling recurs across otherwise unrelated designs. A ROS-generating, arachidonic-acid-supplying nanosystem links immunogenic ferroptosis to IFN-γ-driven ACSL4 upregulation, cooperating with delivered lipid substrate to amplify peroxidation (143), while a MOF@COF nanovaccine couples ROS generation and glutathione depletion to M1 polarization and CD8^+^ priming, with IFN-γ reportedly reinforcing macrophage state and inducing secondary ferroptosis (144). Because ferroptotic death alone is a poor inducer of dendritic cell maturation, several platforms supply the missing adjuvant signal: a stimuli-responsive liposome bearing a di-arachidonyl phospholipid and CpG triggers DC maturation and CD8^+^ IFN-γ that suppresses SLC7A11 and synergizes with PD-L1 blockade (145).

Checkpoint integration and targeted delivery further extend this circuit. Hemin blocks the TIGIT/PVR interaction and induces ferroptosis, restoring CD8^+^ IFN-γ that synergizes to drive tumor ferroptosis, including in an anti-PD-1-resistant model (146). Vesicle-based vectors enhance tumor selectivity: NK-cell-exosome/RSL3 hybrid liposomes co-deliver FASL, IFN-γ, and a GPX4 inhibitor to glioma (147), and engineered bacterial outer membrane vesicles cross the blood–brain barrier to drive CD8^+^ IFN-γ-mediated, system-Xc^-^/GPX4-dependent ferroptosis, sensitizing temozolomide-resistant glioblastoma (148).

Conceptually, these systems aspire to a closed loop in which transient activation becomes a self-renewing antitumor pressure, which is the explicit design goal of mutually reinforced ferroptosis–immunotherapy strategies (166). However, the demonstrated evidence is one of synergy in largely preclinical models, not proof of a perpetually self-sustaining circuit; indeed, nonselective ferroptosis can itself be immunosuppressive (147), underscoring why targeting and adjuvant pairing are treated as central rather than incidental approaches. Read through the IFN-γ Response Cycle, these platforms represent attempts to weld execution back to initiation, closing the loop that the broader synthesis now formalizes.

#### 5.5.3 Microenvironment reprogramming and physical/chemo combinations

The execution of IFN-γ-centered immunity is contingent on context; even a competent effector response fails in a tumor whose stroma, metabolism, vasculature, and checkpoint tone render it immune-excluded. Therefore, a large body of engineering targets not the effector but the ecosystem, seeking to convert immune-restrictive (“cold”) tumors into states permissive to productive immunity, often using IFN-γ both as the endpoint that reports permissiveness and as a deliverable input that helps create it. Physical modalities provide a route for local ignition of the reaction. Ultrasound-based platforms combine sonodynamic immunogenic cell death (ICD) with cavitation-mediated stromal disruption to open dense HCC to T-cell infiltration and IFN-γ (150), or use focused ultrasound to activate bacterially encoded IFN-γ along with doxorubicin-driven ICD (151). Photothermal and photodynamic approaches similarly elicit ICD, DC maturation, and IFN-γ, including the abscopal control of untreated lesions (152). Notably, one photothermal design also neutralizes PD-L1 and IDO1, which it provokes (153), a reminder that ablation alone can trigger the cycle’s own restraint.

The second axis dismantles the metabolic and structural barriers. Because lactate sustains an immunosuppressive milieu, inhibiting its excretion via GLUT1 blockade, paired with PD-1/PD-L1 blockade to offset IFN-γ-induced PD-L1, reduces suppressive macrophages and Tregs in glioblastoma (154), while CRISPR-mediated PKM2 knockdown curtails the Warburg effect, lowers HIF-1α and PD-L1, and shifts cytokines toward IFN-γ and IL-12 in head and neck models (155). Stromal and vascular conditioning act analogously: the matrix proteoglycan decorin enhances CD8^+^ infiltration and IFN-γ in a β2M- and CD8-dependent manner and augments anti-PD-1 efficacy (156). VEGFR2-targeted vaccination with paclitaxel-coupled vascular modulation enhances CD8^+^ IFN-γ responses (157).

Chemotherapy, microbial therapy, and oncolytic platforms converge on the same endpoint from different directions. IFN-γ can be exploited directly to raise MHC-I and sensitize “cold” prostate cancer to chemotherapy (158), or co-delivered with doxorubicin to amplify ICD and MHC-I expression in breast tumors (159). Bacterial outer membrane vesicles enable tumor cell self-blockade of PD-L1 while recruiting lymphocytes that secrete IFN-γ (160). An *in situ* ICD-amplifying payload sensitizes KRAS-mutant colorectal tumors to checkpoint blockade (161). Oncolytic reovirus combined with a CEA vaccine and PD-1/PD-L1 inhibition lowers regulatory T cells while raising cytotoxic IFN-γ-associated activity (162). What unifies these mechanistically disparate interventions is an endpoint, not a pathway; each appears to shift the tumor toward an IFN-γ-permissive state in which immunity, endogenous or induced, has a higher probability of becoming productive. This shared destination should not be interpreted as a shared mechanism, and the evidence remains preclinical. Still, framed by the IFN-γ Response Cycle, microenvironment reprogramming conditions the very stage on which initiation, execution, constraint, and evolution play out, the integration to which the concluding synthesis now turns.

## 6 Part VI. Reading the cycle (sensors): IFN-γ as a predictive and pharmacodynamic biomarker

### 6.1 Transcriptional and longitudinal signatures

#### 6.1.1 Validated IFN-γ transcriptional signatures predicting checkpoint response

The concentration of a cytokine says little about whether its program ran. IFN-γ transcriptional signatures address this by reading not the ligand but its integrated downstream consequences across tumor and immune compartments, offering a system-level proxy for whether the IFN-γ Response Cycle is actively executing rather than merely whether its parts are present. Because the biomarker literature uses *prognostic* and *predictive* inconsistently, we apply three labels throughout this Part and in **Table 3**. Prognostic denotes an association with outcome that has been shown to hold irrespective of treatment, either because untreated patients were included or because the association was consistent across treatment arms. Response-associated denotes an association with response or outcome observed only among treated patients; because such designs contain no untreated comparator, a response-associated marker cannot be formally distinguished from a prognostic one. Pharmacodynamic denotes a measure of on-treatment biology rather than of outcome. We reserve *predictive* for its strict meaning, a demonstrated biomarker-by-treatment interaction, and note at the outset that no IFN-γ biomarker considered here meets that standard, since nearly all derive from single-arm or uniformly treated cohorts. **Table 3** collates the readouts considered across this section by what they sample and in which compartment, alongside their evidence class as defined above and the principal limitation of each. Across settings, these signatures converge on the same biology. In melanoma, T-cell infiltration and IFN-γ-signaling signatures corresponded most closely with checkpoint response, with a reciprocal fall in cell-cycle and WNT programs, and an *in vitro* conserved IFN-γ transcriptome that failed only when tumor cells carried IFNGR alterations, linking the signature to intact pathway execution (167). A pan-tumor T-cell-inflamed gene-expression profile, built across hundreds of patients and multiple cancers, captured IFN-γ-responsive genes spanning antigen presentation, chemokines, and cytotoxicity and predicted benefit, though it was necessary but not always sufficient for response (19). Compact CD8^+^-associated signatures enriched for cellular responses to IFN-γ and antigen processing carry prognostic weight in melanoma and are additionally response-associated in checkpoint-treated cohorts (168), and analogous IFN-γ-related signatures show the same dual behavior in bladder cancer across real-world cohorts (169). That a signature is both prognostic and response-associated is itself informative: it indicates that the marker reports baseline tumor–immune biology as well as behavior under therapy, and therefore that its treated-cohort association cannot be attributed to treatment specificity.

**Table 3:** Reading the cycle, IFN-γ as a predictive and pharmacodynamic biomarker across compartments.

Biomarker	Type	Compartment	Tumor/cohort	Evidence class	Key limitation	Ref.
A. Validated transcriptional IFN-γ signatures — reads: presence and dominance of an IFN-γ-driven inflammatory state; does not resolve node						
T-cell-infiltration + IFN-γ-signaling signature	*Transcriptional*	Tumor (bulk)	Melanoma (101 pts, anti-PD-1 ± anti-CTLA-4)	Response-associated	Correlative; the conserved IFN-γ transcriptome failed only when tumor cells carried IFNGR alterations, so the readout presupposes an intact pathway.	(167)
Pan-tumor T-cell-inflamed gene-expression profile	*Transcriptional*	Tumor (bulk)	Pan-tumor (multiple cancers)	Response-associated	Necessary but *not always sufficient* for response: a high score does not guarantee benefit.	(19)
9-gene CD8^+^/IFN-γ co-expression signature	*Transcriptional*	Tumor (bulk)	Melanoma	Prognostic; response-associated	Derived from TCGA/GEO by deconvolution; correlative and in need of prospective validation.	(168)
IFN-γ-related prognostic signature	*Transcriptional*	Tumor (bulk)	Bladder (TCGA + 4 ICI cohorts)	Prognostic; response-associated	Real-world/retrospective cohorts; associative rather than causal.	(169)
IFN-γ: immunosuppression signature ratio	*Transcriptional (ratio)*	Tumor (bulk)	Melanoma + gastric	Response-associated	A derived ratio that is improved over established profiles but remains correlative. Captures the dominance of activation over suppression, not absolute activity.	(170)
B. Neoadjuvant & longitudinal signatures — reads: direction and magnitude of change over time; direction alone is not node-diagnostic						
Baseline IFN-γ score (RFS)	*Transcriptional (baseline)*	Tumor (bulk)	Macroscopic stage III melanoma (adjuvant)	Prognostic (association present in untreated patients)	Validated in one external cohort but *not another*; high score predicted longer RFS in treated *and untreated* patients, blurring prognostic vs. predictive value.	(171)
IFN-γ score + tumor mutational burden	*Transcriptional + TMB*	Tumor (bulk)	Stage III melanoma (neoadjuvant ipi+nivo)	Response-associated	Small neoadjuvant trial cohorts (OpACIN/OpACIN-neo); a combined two-marker readout — near-uniform response only when both high.	(172)
Prospective baseline IFN-γ stratification algorithm	*Transcriptional (algorithm)*	Tumor (bulk)	Stage III melanoma (neoadjuvant)	Response-associated (prospective stratification)	The HDAC-inhibitor (domatinostat) arm was deployed prospectively, but it raised IFN-γ added toxicity without efficacy at the doses tested.	(173)
Early on-treatment adaptive immune signature	*Transcriptional (longitudinal)*	Tumor (serial)	Metastatic melanoma	Response-associated	Requires serial biopsies; more predictive than pretreatment features but exposes rather than resolves CTLA-4-vs-PD-1 differences and resistance routes.	(174)
Baseline CD8^+^ IFN-γ signature (on-treatment dynamics)	*Transcriptional (scRNA, longitudinal)*	Tumor (single-cell)	Gastric (neoadjuvant immunochemotherapy)	Response-associated	A falling on-treatment IFN-γ readout is ambiguous; it may reflect productive resolution *or* the onset of gating/escape and cannot be assigned to either.	(20)
C. Protein, cellular & circulating biomarkers — reads: evidence of active IFN-γ signaling, sampled across compartments						
Baseline serum IFN-γ	*Protein (circulating)*	Blood (serum)	Mixed/pan-cancer (retrospective)	Response-associated	Associated with disease control and OS but *inconsistently across endpoints*; a single circulating value does not necessarily mirror intratumoral biology.	(175)
Pretreatment cytokine panel (IFN-γ + IL-6 + IL-10)	*Protein (circulating panel)*	Blood (serum)	Advanced melanoma (phase 2 nivolumab)	Response-associated	Informative in a panel context rather than in isolation; single-arm phase 2 cohort study.	(176)
PBMC IFN-γ, IFN-γ/IL-10 ratio, CD4^+^IFN-γ^+^ fraction	*Cellular*	PBMC	Advanced melanoma	Response-associated	Small cohort; peripheral cellular readouts may not mirror the tumor compartment.	(177)
Inducible IFN-γ capacity on ex vivo stimulation	*Functional*	PBMC (ex vivo)	Metastatic melanoma	Response-associated	Unstimulated serum cytokines were uninformative; the assay reports functional *capacity*, not spontaneous *in vivo* activity.	(178)
Intratumoral IFNG transcript	*Transcriptional (tissue)*	Tumor (tissue)	NSCLC + melanoma	Response-associated	Open question whether it outperforms PD-L1 staining, which is a static, local measure.	(179)
Microenvironmental IFN-γ protein + CD8^+^ TIL density	*Protein (tissue)*	Tumor (tissue)	Urothelial (pembrolizumab, n=26)	Response-associated (pembrolizumab-treated cohort only)	Small cohort; a static, local snapshot — worst outcomes only where both were low.	(180)
IFN-γ-induced IP-10 (CXCL10) release — “IP-10 score”	*Protein (surrogate)*	Ex vivo/tissue	HNSCC (cetuximab + pembrolizumab)	Response-associated	Downstream chemokine *surrogate* for IFN-γ activity rather than the cytokine itself; small cohort.	(181)
D. Integrated, contextual & computational models — reads: the decoding hierarchy, IFN-γ read against antigenicity, presentation & suppression						
Integrated clinical + genomic + transcriptomic (MHC-I/II) model	*Integrated/computational*	Tumor (multi-omic)	Metastatic melanoma (n=144)	Response-associated	Correlative and cohort-bound; TMB lost coherence once the subtype was accounted for, and the predictors shifted after prior CTLA-4 exposure.	(182)
Tumor MHC-I loss vs MHC-II + IFN-γ signature	*Integrated (protein + signature)*	Tumor (tissue)	Untreated metastatic melanoma	Response-associated	Distinguishes anti-CTLA-4 from anti-PD-1 resistance; therefore, the predictive value is presentation-layer- and agent-specific, not a single axis.	(183)
IFNGR/JAK/STAT competence + antigenicity + execution	*Integrated (pathway)*	Tumor (cell lines)	Mucosal melanoma	Response-associated	Intact signaling and neoantigens do not guarantee execution; within-pathway heterogeneity is invisible to any presence/absence biomarker.	(184)
Organ-site IFN-γ level + stromal context (fibroblast/TGF-β)	*Integrated/contextual*	Tumor (bulk + IHC)	Mucosal melanoma (101 tumors)	Response-associated	Infiltration without permissiveness does not equal response; IFN-γ levels vary by anatomical site — correlative.	(64)
ML Differential-Phenotype Immune Score (TPX2)	*Integrated/computational*	Tumor (multi-omic)	Lung adenocarcinoma	Prognostic; response-associated	A machine learning construct that is correlative and cohort-bound, nominating rather than proving the proliferation–presentation link.	(185)
E. Spatial & in-situ IFN-γ sensors — reads: the spatial context—where productive engagement occurs (largely preclinical)						
IFN-γ immuno-PET tracer	*Spatial (imaging)*	*In situ* (tumor)	Mouse mammary (HER2/neu vaccine)	Pharmacodynamic	Preclinical; the signal fell to background in PD-1^+^ anergic infiltrates, distinguishing active from inert immunity. Surface localization is an observation, *not proof*, of receptor engagement.	(186)
DNA-origami plasmonic nanoantenna (IFN-γ + TNF-α)	*Spatial/functional (biosensor)*	Ex vivo/in situ	Tumor-bearing mice	Pharmacodynamic	Preclinical; validated against ELISA in a CRS-monitoring context rather than as a response-predictor.	(187)
F. Functional ex vivo & cross-species platforms —reads: the cycle’s operational state under challenge						
Patient-derived micro-tumor platform (IFN-γ + GzmB + CXCL9/11)	*Functional (ex vivo)*	Ex vivo (patient)	Patient-derived micro-tumors	Pharmacodynamic (ex vivo; clinical correlation not established)	It observes responsiveness rather than predicting it from surrogates; the correlation with actual clinical outcomes *remains to be established* (conference abstract).	(188)
Functional IFN-γ response (corticosteroid effect)	*Functional (cross-species)*	PBMC (ex vivo)	Canine cancer (comparative)	Pharmacodynamic	Highly individual results; comparative oncology model — treatment history reshapes competence that static baselines miss.	(189)
Equinized anti-PD-L1 IFN-γ/IL-2 output	*Functional (cross-species)*	PBMC (*in vitro*)	Equine (*in vitro*)	Pharmacodynamic	*In vitro* characterization only; cytokine output used as a surrogate index of checkpoint blockade activity, not validated *in vivo*.	(190)

Biomarkers are arranged by what they sample and where, not by assay platform. *Transcriptional signatures* (A) read the integrated downstream consequences of IFN-γ, a system-level proxy for whether the cycle is executing, while neoadjuvant and longitudinal designs (B) convert that snapshot into a trajectory, establishing that IFN-γ-associated activity changes under therapy without yet resolving whether a given change reflects resolution or escape. *Protein, cellular, and circulating measures* (C) attempt to capture execution as it happens across blood, PBMC, and tissue, and *integrated computational models* (D) show that fragments acquire predictive meaning only when read against antigenicity, presentation competence, and suppression. *Spatial sensors* (E) add the dimension of *where* engagement occurs, and *functional ex vivo and cross-species platforms* (F) interrogate the cycle under challenge. A theme runs through the Key-limitation column: every readout is correlative and context-dependent, none is a direct measure of cytokine activity, and convergence across compartments, not any single value, is the more faithful estimate of whether IFN-γ-driven immunity is operating; none of these readouts, individually or in combination, has yet been shown to assign a tumor to a defined stage of the cycle, which remains an objective rather than a demonstrated capability. Evidence classes follow the definitions given in Section 16.1. Prognostic requires an association demonstrated to hold irrespective of treatment; response-associated denotes an association observed only within treated patients, which no single-arm design can separate from a prognostic effect; pharmacodynamic denotes a measure of on-treatment biology rather than of outcome. No entry in this table meets the strict definition of a predictive biomarker, which requires a demonstrated biomarker-by-treatment interaction, and we have therefore not used that label.

The fact that these composites outperform isolated markers reflects what they index: immune functionality, not immune presence. The “necessary-but-not-sufficient” gap is instructive, since an IFN-γ program can be simultaneously productive and counterbalanced by adaptive resistance. Quantifying this balance directly, the ratio of an IFN-γ signature to an immunosuppression signature improved prediction over multiple established profiles across several melanoma and gastric cohorts (170), suggesting that what matters is not activation alone but its dominance over suppression. Interpreted through the cycle, these signatures indicate an IFN-γ-associated inflammatory state and, in the ratio-based formulation, the relative balance between an IFN-γ signature and an immunosuppression signature, with a higher ratio associated with a better anti-PD-1 response (170). However, they do not localize a tumor to a particular node of the cycle. This limitation is structural rather than technical: interferon-stimulated programs can arise in both cancer and immune cells, where tumor-cell and immune-cell interferon responses can have opposing relationships with checkpoint efficacy (10). A bulk-tissue signature therefore aggregates signals from these cellular compartments. The T cell–inflamed GEP contains IFN-γ-responsive genes related to antigen presentation, chemokine expression, cytotoxic activity, and adaptive immune resistance (19), illustrating how a single inflammatory signature can encompass both immune activation and counter-regulatory programs. These signatures therefore remain correlative, context-dependent proxies for an inflamed state rather than direct measurements of IFN-γ activity or of cycle position. Because they are generally derived from static tissue measurements, they also do not by themselves resolve how this inflammatory state changes during therapy, motivating the longitudinal monitoring considered next.

#### 6.1.2 Neoadjuvant and longitudinal signatures

A single biopsy fixes what is, in fact, a moving process. Neoadjuvant and longitudinal designs exploit the one setting where serial tumor tissue is routinely available, allowing IFN-γ-associated signatures to be read as trajectories rather than snapshots, and thereby to approximate the kinetic transitions the IFN-γ Response Cycle predicts. At baseline, IFN-γ scores carry demonstrable prognostic weight, and the distinction from predictive value is instructive. In macroscopic stage III melanoma, a high IFN-γ signature was associated with longer recurrence-free survival in patients who did and did not receive adjuvant therapy, validating in one external cohort but not another; the authors concluded that the signature was prognostic in both groups (171). Because the association held in the absence of treatment, it reports baseline tumor–immune biology rather than benefit specific to checkpoint blockade. In the neoadjuvant OpACIN/OpACIN-neo trials, a high IFN-γ score combined with a high mutational burden corresponded to a near-uniform pathologic response, versus far lower rates when both were low (172). Such scores can be deployed prospectively: a baseline IFN-γ algorithm was used to stratify patients to distinct neoadjuvant regimens, even though the HDAC-inhibitor arm intended to raise IFN-γ added toxicity without efficacy at the doses tested (173).

The greater information, however, lies in change. Early on-treatment adaptive immune signatures proved more predictive than pretreatment features and exposed differential microenvironmental effects of CTLA-4 versus PD-1 blockade along with candidate resistance mechanisms (174). In gastric cancer, a high baseline CD8^+^ IFN-γ signature predicted response to neoadjuvant immunochemotherapy; however, the same signature declined across cell types during treatment while exhaustion was suppressed (20), a reminder that a falling IFN-γ readout may equally reflect productive resolution or the onset of gating and escape, and cannot by itself be assigned to either. Read through the cycle, these trajectories represent a step toward, rather than an achievement of, the classification the framework would require. The gastric data make the difficulty concrete: the IFN-γ signature decreased during neoadjuvant therapy alongside suppression of the CD8^+^ T-cell exhaustion signature, so a change in either readout alone cannot establish whether the tumor has undergone productive resolution or entered resistance without additional measurements that distinguish these states (20). Therefore, the direction of change alone is not diagnostic of cycle position. Longitudinal studies show that immune signatures measured during treatment can differ from pretreatment states and can provide information about response and resistance that baseline measurements do not (174), establishing the value of trajectory without by itself resolving the underlying state. Resolving this ambiguity would require measurements that distinguish the cellular and functional components captured by bulk or composite signatures, such as compartment-resolved single-cell or spatial profiling paired with independent markers of immune restraint; such an integrated longitudinal approach has not yet been established for this setting.

### 6.2 Protein, cellular, and integrated biomarkers

#### 6.2.1 Protein, cellular, and circulating IFN-γ biomarkers

Transcriptional signatures describe the program, protein, cellular, and circulating biomarkers attempt to capture its execution as it happens, and across compartments rather than in tissue alone. Each class samples a different facet of productive IFN-γ activity, and their shared lesson is that benefit tracks evidence of functional signaling more than the static presence of immune cells. In the circulation, baseline serum IFN-γ has been associated with improved disease control and overall survival under checkpoint blockade, albeit inconsistently across endpoints (175), and pretreatment cytokine panels in which IFN-γ rises alongside IL-6 and IL-10 in responders indicate that the cytokine is informative in context rather than in isolation (176). Cellular assays add specificity: in peripheral blood mononuclear cells (PBMCs), higher IFN-γ expression and a higher IFN-γ/IL-10 ratio, together with an expanded CD4^+^IFN-γ^+^ fraction, distinguished responders (177), an activation-to-suppression reading that echoes the transcriptomic balance metrics. Functional monitoring sharpens this further; unstimulated serum cytokines were uninformative, whereas the capacity to mount IFN-γ upon ex vivo stimulation and its trajectory through early treatment correlated with progression-free survival (178). Cellular readouts in blood extend this logic beyond the cytokine itself: interferon-stimulated Ly6E^hi^ neutrophils, induced by tumor-intrinsic STING signaling and therefore reflecting type I as much as type II interferon input, predicted anti-PD-1 response in mice and in patients with NSCLC and melanoma and could sensitize otherwise resistant tumors in part through IL12b-dependent T-cell activation (191); and in advanced NSCLC, blood eosinophils together with circulating IFN-γ were associated with both immune-related adverse events and longer progression-free survival (61)​, illustrating that peripheral markers of benefit and of toxicity can be difficult to separate.

Tissue- and surrogate-level measures anchor these systemic readouts to tumors. Intratumoral IFNG transcripts are associated with longer survival in NSCLC and melanoma, prompting the question of whether they outperform PD-L1 staining (179). IFN-γ protein in the urothelial microenvironment, combined with CD8^+^ tumor-infiltrating lymphocyte (TIL) density, was independently associated with outcomes, with the worst outcomes where both were low (180). Because this cohort consisted entirely of patients receiving pembrolizumab, this is a response-associated rather than a prognostic finding, notwithstanding the terminology used in the source. Ex vivo assays have linked IFN-γ-induced IP-10 (CXCL10) release to improved outcomes and yielded a response-associated ‘IP-10 score’ in head-and-neck cancer (181). No single compartment is complete in the model. Circulating measures are accessible but do not necessarily mirror intratumoral biology; tissue measures are local but static; and functional assays capture capacity rather than spontaneous activity. Read through the cycle, each of these samples productive execution from a different angle, and their convergence, rather than any single value, offers a more faithful estimate of whether IFN-γ-driven immunity is operating, although convergence across compartments still indicates an active state rather than a stage within it.

#### 6.2.2 Integrated, contextual, and computational prediction models

If single biomarkers capture fragments of IFN-γ biology, the recurring finding of integrative studies is that the fragments acquire predictive meaning only when combined. For instance, the tumor mutational burden loses coherence once the melanoma subtype is accounted for, whereas models integrating clinical, genomic, and transcriptomic features, prominently those of MHC-I and MHC-II antigen presentation, predict selective resistance to anti-PD-1 therapy, with the relevant predictors shifting after prior CTLA-4 exposure (182). Antigen presentation emerges as a layer that reroutes the same IFN-γ context toward different therapies. Loss of tumor MHC-I predicts primary resistance to anti-CTLA-4 but not to anti-PD-1. In contrast, MHC-II expression coupled with IFN-γ-mediated signatures predicts anti-PD-1 response, implying that the benefit of combination blockade reflects distinct antigen-presentation requirements rather than a single activation axis (183). Responsiveness is a dimension separate from signaling competence. In mucosal melanoma, most lines retained intact IFNGR/JAK/STAT signaling and immunogenic neoantigens, yet only a subset executed IFN-γ programs while poorly differentiated cells remained refractory (184), a within-pathway heterogeneity invisible to any presence/absence biomarker.

The context extends to the ecosystem and anatomy. Across mucosal melanoma, IFN-γ signature levels varied by organ site, and tumors that were immune-infiltrated yet ICB-unresponsive carried elevated fibroblast, macrophage, and TGF-β signals, indicating that infiltration without permissiveness does not equal response (64). Machine learning frameworks formalize this multidimensionality: an integrated immune score capturing proliferation-driven suppression stratified survival across cohorts and nominated TPX2 as a link between proliferative signaling and impaired antigen presentation, while IFN-γ-dominant and inflammatory states of comparable checkpoint sensitivity diverged in prognosis (185). All in all, these models operationalize the decoding hierarchy: IFN-γ acquires predictive value only when read against antigenicity, presentation competence, immune composition, and suppressive or proliferative constraints. They remain correlative and cohort-bound rather than causal proofs, but they reframe IFN-γ as a context-dependent signal, the central claim the concluding synthesis now consolidates.

### 6.3 Spatial and functional pharmacodynamic readouts

#### 6.3.1 Spatial and *in-situ* IFN-γ sensors

The circulating and bulk biomarkers report the amount of IFN-γ activity in a tumor but not where it is occurring, and localization may be precisely where biological meaning resides. Spatial imaging and *in situ* biosensing aim to convert IFN-γ from an inferred process into a directly observed one. Noninvasive imaging makes the clearest case that location and function are coupled. An IFN-γ immuno-PET tracer detected cytokine elevation *in situ* after vaccination, with uptake inversely correlating with tumor growth; critically, in a model where CD8^+^ T cells infiltrated but upregulated PD-1, the tracer signal fell to the background despite the cells’ presence, distinguishing active from anergic immunity that infiltration counts alone would conflate (186). The tracer appeared to localize IFN-γ sequestered on tumor-cell surfaces, plausibly in receptor complexes, an observation, not proof, that the imaged signal marks sites of receptor engagement.

Biosensing aims to achieve sensitivity and multiplexing. Surface-enhanced Raman scattering platforms have been reported to detect checkpoint proteins and cytokines, including IFN-γ, at very low concentrations, and coupled with machine learning, to classify immune phenotypes in preclinical models (192). Programmable DNA-origami plasmonic nanoantennas extend this to logic-gated, simultaneous quantification of IFN-γ and TNF-α, validated against ELISA in treated tumor-bearing mice (187). These preclinical tools measure several analytes simultaneously, a prerequisite for reading IFN-γ within its signaling context rather than in isolation. In summary, such technologies begin to operationalize the spatial-context dimension, indicating not merely whether IFN-γ is present but where productive engagement may be occurring. They remain early and context-bound, yet extend biomarker assessment into the spatial dimension that the concluding framework now integrates with time and evolution.

#### 6.3.2 Functional ex vivo and cross-species translational platforms

Most biomarkers infer responsiveness from fixed molecular states. Functional platforms perturb the system and observe its behavior, treating IFN-γ output as a readout of immune competence, rather than a static feature. This distinction is important because the operational status of the IFN-γ Response Cycle is, by definition, a property revealed under challenge. Patient-derived ex vivo systems provide concrete evidence. A microtumor platform that preserves the tumor microenvironment exposed tissue to checkpoint inhibitors and a bispecific T-cell engager and scored immune cell expansion and tumor killing directly; effective responses were accompanied by coordinated upregulation of IFN-γ, IL-2, granzyme B, perforin, and CXCL9/CXCL11, and tracked with immune cell density and, partially, with PD-L1 status (188). Such assays observe responsiveness rather than predict it from surrogates; however, as the authors note, their correlation with actual clinical outcomes remains to be established.

Comparative oncology provides complementary models for spontaneously arising tumors. In dogs, prior corticosteroid exposure altered PBMC composition and the functional IFN-γ response to PD-1/PD-L1 blockade and IL-12, with highly individual results, providing evidence that treatment history can reshape immune competence in ways that static baselines may miss (189). In horses, an equinized anti-PD-L1 antibody increased IFN-γ and IL-2 levels in PBMC cultures, using cytokine output as an *in vitro* index of checkpoint blockade activity (190). Across species and systems, IFN-γ is the functional signature of a productively responsive immune compartment. These platforms remain preclinical and have not yet been validated against clinical endpoints; however, they begin to prospectively interrogate the cycle’s operational state, closing the loop between measurement, prediction, and intervention that the following synthesis (Section 18.3) makes explicit.

#### 6.3.3 From complexity to a decision framework: reading the cycle’s operational state

IFN-γ should not be interpreted as a stand-alone biomarker. Pretreatment IFN-γ-related transcriptional signatures have been associated with response to PD-1-based therapy (19, 20), yet longitudinal analyses also show that these programs may decline during treatment despite favorable pathological response (20). Their interpretation therefore depends on additional features, including antigen-presentation capacity, IFN-γ-pathway integrity, prior treatment context, and the composition of the tumor microenvironment (64, 182, 183, 185). We therefore propose a three-profile framework for interpreting the operational state of the IFN-γ cycle; these profiles are conceptual rather than clinically validated categories.

A *productive profile* is characterized by preserved IFN-γ signaling and antigen-presentation machinery together with evidence of immune-effector activity. The biological relevance of these components is supported by studies linking intact IFN-γ signaling to checkpoint sensitivity (6, 7), MHC-I and MHC-II expression to differential responses to immune-checkpoint blockade (182, 183), and coordinated effector outputs such as IFN-γ, granzyme B, and CXCL9/CXCL11 to ex vivo immunotherapy responses (188). Such a profile is therefore consistent with ongoing immune engagement, although it does not by itself define a therapeutic decision.

A second, *counter-regulated profile*, describes tumors in which inflammatory activity coexists with suppressive or exclusionary features. Immune-infiltrated but checkpoint-unresponsive mucosal melanomas have shown enrichment of macrophage-, fibroblast-, and TGF-β-associated programs (64), while immune-state analyses in lung adenocarcinoma identified heterogeneity involving PD-L1 expression, T-cell dysfunction, immune exclusion, and other suppressive features (185). These observations indicate that a strong inflammatory or IFN-γ-associated signal does not necessarily imply productive antitumor immunity.

An *escape-associated profile* is supported when direct evidence demonstrates impaired tumor-cell IFN-γ signaling or antigen presentation. Acquired JAK1/JAK2 loss-of-function alterations and B2M disruption have been identified in melanomas resistant to PD-1 blockade (6), while loss of IFN-γ-pathway genes has been associated with resistance to CTLA-4 blockade (7). Such alterations should therefore be distinguished from a merely low IFN-γ signal. Importantly, pathway loss does not establish universal therapeutic refractoriness; preclinical work has identified context-specific vulnerabilities in IFN-γ-pathway-deficient melanoma (108).

This framework also clarifies why changes in IFN-γ over time require contextual interpretation. A declining IFN-γ-related signature may occur during successful therapy (20), whereas progression may coincide with acquired defects in IFN-γ signaling or antigen presentation (6). Accordingly, longitudinal IFN-γ measurements should be interpreted together with tumor trajectory and, where feasible, direct assessment of pathway and antigen-presentation integrity. Future biomarker strategies should therefore integrate molecular, cellular, spatial, and functional information (64, 182, 185, 188–190). This framework remains hypothesis-generating and requires prospective longitudinal validation before it can support clinical decision-making.

### 6.4 Limitations and unresolved questions

Several constraints temper the claims made here. First, the temporal architecture of the cycle is an interpretive framework rather than a measured sequence of events. We are unaware of a study that has quantified the productive, restraining, and escape arms as separately quantified states within a single tumor over time; the ordering described here is inferred from the differing kinetics, durability, and cellular requirements of programs that are, when directly measured, co-induced (4, 5, 11). Several mechanisms grouped here for exposition were established in distinct and non-overlapping systems. Therefore, we have marked where links between them represent our proposed synthesis rather than demonstrated pathways, most explicitly for the relationship between IFN-γ-driven metabolic restriction and IFN-γ-driven ferroptosis (Section 4.2). Second, the cycle’s central causal assertion rests on indirect evidence. The fact that IFN-γ enriches resistant clones rather than directly mutagenizing them is inferred from clonal cooperation and selection studies (5) and from the recurrent loss of IFN-γ-pathway and antigen-presentation genes in relapsing tumors (6, 7). However, no study has tracked this selection prospectively and longitudinally in patients, leaving the enrichment-versus-induction distinction supported in principle rather than demonstrated. Third, the evidence base is unbalanced, as set out in Section 2: the interventional evidence is almost entirely murine and heavily weighted toward melanoma, whereas the human evidence is predominantly observational (10, 16). Context dependence is documented and examined in Section 6.6: IFN-γ-driven metabolic restriction curbed growth in melanoma but not colon carcinoma (18), single-cell prognostic associations ran in opposite directions across tumor types (15), IFN-γ signature levels varied by anatomical site even within one histology (64), and the same lineage decoded IFN-γ oppositely in the liver and the intestine (54, 63). Therefore, generalization across cancers and clinics is provisional. A related attribution gap concerns toxicity: irAEs are associated with greater clinical benefit from checkpoint blockade (59, 60), and circulating IFN-γ has been associated with both irAEs and favorable outcomes in an NSCLC cohort (61). These observations support overlapping inflammatory biology but do not establish a shared IFN-γ source or mechanism of action. No clinical study has determined whether IFN-γ contributes to tissue toxicity originating in the tumor microenvironment or is produced locally in affected normal tissues, limiting the inference that efficacy and toxicity are mechanistically inseparable. Fourth, biomarker logic is correlative. IFN-γ signatures do not measure cytokine concentration or causal activity, remain necessary but not sufficient for response (19), and their decline under therapy is ambiguous, plausibly reflecting either productive resolution or incipient escape (20). More fundamentally, a bulk signature aggregates interferon-stimulated genes whose associations with outcomes run in opposite directions depending on the compartment expressing them (10); therefore, it measures an inflammatory state rather than a position within the cycle. The node assignments used throughout this review are interpretive placements of what each assay plausibly reports, not validated classifications, and establishing them would require compartment-resolved longitudinal and spatial measurements that have not yet been performed. A related constraint is the classification of biomarkers. Because almost all of the studies reviewed enrolled uniformly treated cohorts, they established that a marker is associated with outcome under therapy but cannot distinguish that from a general prognostic effect; where untreated patients were included, the association frequently persisted (171). Therefore, IFN-γ biomarkers should be regarded as prognostic or response-associated rather than validated predictors of the benefits of checkpoint blockade. Fifth, the framework is qualitative: although outputs are dose- and duration-dependent (4, 10), the quantitative thresholds that would convert these determinants—signal intensity, duration, recipient cell, spatial organization, and network context—into a predictive instrument are undefined.

Finally, therapeutic corollaries are largely unvalidated clinically; signal-uncoupling and pathway-exploiting strategies remain preclinical (107, 108), and the single trial pairing IFN-γ with checkpoint blockade established tolerability while inducing PD-L1 (17). Whether interrupting feedback delays resistance, whether cumulative IFN-γ productivity is measurable in patients, and which tumors benefit most from decoupling remain open.

## 7 Conclusion

IFN-γ has long been recognized as a context-dependent mediator of antitumor immunity that can also contribute to immune escape. The evidence reviewed here supports a more integrated interpretation, but not a universal causal sequence. IFN-γ can directly inhibit tumor-cell growth and enhance antigen presentation, while sustained or low-level signaling can also induce counter-regulatory programs such as PD-L1 and, in heterogeneous tumors, create conditions in which IFN-γ-insensitive clones gain a selective advantage (4, 5). Clinical studies further show that defects in IFN-γ signaling or antigen-presentation pathways can accompany resistance to immune-checkpoint blockade, including acquired JAK1/JAK2 or B2M alterations after anti-PD-1 therapy and IFN-γ-pathway defects associated with primary resistance to anti-CTLA-4 therapy (6, 7).

These findings argue against treating the antitumor and resistance-associated effects of IFN-γ as completely independent phenomena. Instead, they support a context-dependent model in which the same pathway that contributes to immune control may also generate adaptive inhibitory signals or selective pressures that favor resistant subpopulations. Importantly, this model should not be interpreted to mean that effective IFN-γ signaling inevitably produces resistance; the outcome depends on signaling intensity and duration, tumor heterogeneity, pathway integrity, and the surrounding immune context (4, 5).

This reframing has a testable therapeutic implication. Rather than simply increasing or suppressing IFN-γ activity, future strategies should determine whether beneficial outputs, such as growth inhibition and antigen presentation, can be preserved while limiting adaptive inhibitory programs and the expansion of IFN-γ-insensitive clones. It also suggests that tumor responsiveness to IFN-γ may be most informative when assessed dynamically during treatment rather than as a fixed baseline property. At present, however, this remains a hypothesis-generating framework rather than an established therapeutic principle. A phase I study combining recombinant IFN-γ with nivolumab demonstrated acceptable safety but only modest exploratory efficacy; IFN-γ exposure was also associated with increased PD-L1 expression on circulating monocyte subsets, while tumor PD-L1 increased only in a subset of evaluable patients (17). The key next step is therefore to define biomarkers that distinguish productive IFN-γ signaling from adaptive inhibition or emerging resistance, and to test prospectively whether those states can guide treatment selection or combination strategies.
